# Systematic identification of CAZymes and transcription factors in the hypercellulolytic fungus *Penicillium funiculosum* NCIM1228 involved in lignocellulosic biomass degradation

**DOI:** 10.1186/s13068-023-02399-9

**Published:** 2023-10-04

**Authors:** Nandita Pasari, Mayank Gupta, Tulika Sinha, Funso Emmanuel Ogunmolu, Syed Shams Yazdani

**Affiliations:** 1 Microbial Engineering Group, International Centre for Genetic Engineering and Biotechnology, Aruna Asaf Ali Marg, New Delhi, India; 2 DBT-ICGEB Centre for Advanced Bioenergy Research, International Centre for Genetic Engineering and Biotechnology, Aruna Asaf Ali Marg, New Delhi, India; 3 Present Address: National Institute of Plant Genome Research, Aruna Asaf Ali Marg, New Delhi, India

**Keywords:** *Penicillium funiculosum* NCIM1228, Genome annotation, CAZymes, Biomass hydrolyzing enzymes, Transcription factors, Regulation

## Abstract

**Background:**

*Penicillium funiculosum* NCIM1228 is a filamentous fungus that was identified in our laboratory to have high cellulolytic activity. Analysis of its secretome suggested that it responds to different carbon substrates by secreting specific enzymes capable of digesting those substrates. This phenomenon indicated the presence of a regulatory system guiding the expression of these hydrolyzing enzymes. Since transcription factors (TFs) are the key players in regulating the expression of enzymes, this study aimed first to identify the complete repertoire of Carbohydrate Active Enzymes (CAZymes) and TFs coded in its genome. The regulation of CAZymes was then analysed by studying the expression pattern of these CAZymes and TFs in different carbon substrates—Avicel (cellulosic substrate), wheat bran (WB; hemicellulosic substrate), Avicel + wheat bran, pre-treated wheat straw (a potential substrate for lignocellulosic ethanol), and glucose (control).

**Results:**

The *P. funiculosum* NCIM1228 genome was sequenced, and 10,739 genes were identified in its genome. These genes included a total of 298 CAZymes and 451 TF coding genes. A distinct expression pattern of the CAZymes was observed in different carbon substrates tested. Core cellulose hydrolyzing enzymes were highly expressed in the presence of Avicel, while pre-treated wheat straw and Avicel + wheat bran induced a mixture of CAZymes because of their heterogeneous nature. Wheat bran mainly induced hemicellulases, and the least number of CAZymes were expressed in glucose. TFs also exhibited distinct expression patterns in each of the carbon substrates. Though most of these TFs have not been functionally characterized before, homologs of NosA, Fcr1, and ATF21, which have been known to be involved in fruiting body development, protein secretion and stress response, were identified.

**Conclusions:**

Overall, the *P. funiculosum* NCIM1228 genome was sequenced, and the CAZymes and TFs present in its genome were annotated. The expression of the CAZymes and TFs in response to various polymeric sugars present in the lignocellulosic biomass was identified. This work thus provides a comprehensive mapping of transcription factors (TFs) involved in regulating the production of biomass hydrolyzing enzymes.

**Supplementary Information:**

The online version contains supplementary material available at 10.1186/s13068-023-02399-9.

## Background

With the increasing world population, there has been a surge in the global demand for generating inexpensive, abundant, and sustainable energy resources [1]. One of the best alternative energy sources to petroleum-based fuels is biofuels produced using renewable resources [2]. Biofuels derived from a renewable resource such as lignocellulosic biomass appear promising as they are abundant and do not compete with food production [3]. However, producing lignocellulose-based second-generation biofuels is challenging due to the recalcitrant nature of the plant cell wall [4]. It comprises cellulose, hemicellulose, and lignin embedded in the pectin matrix [5]. Its complete hydrolysis requires thermo-chemical pre-treatment to loosen up its structure, followed by the action of specialized enzymes which can specifically hydrolyze the β-linkages between glucose moieties [6, 7]. Classical enzymes such as cellulases, hemicellulases, and pectinases hydrolyze the plant cell wall components along with auxiliary activity enzymes, which act synergistically with the classical enzymes to loosen up the lignocellulosic structure, increasing the accessibility of hydrolyzing enzymes [8, 9]. These enzymes are categorized as Carbohydrate Active Enzymes (CAZymes). They are subdivided based on their functions into various families, such as Glycoside Hydrolase (GH), Glycosyl Transferase (GT), Polysaccharide Lyase (PL), Carbohydrate Esterase (CE), and Auxiliary Activity (AA) [10].

Filamentous fungi are natural decomposers and play a dominant role in nutrient cycling in soil [11]. They can thrive on plant biomass by producing a battery of enzymes with different specificities to hydrolyze biomass [12, 13]. Enzyme mixtures produced by fungi are characterized by high productivity and high catalytic efficiency compared to other organisms [12]. They have exceptional secretory capacity, due to which they have been used in industry for a long time. They have proven beneficial to the bioethanol industry for the hydrolysis of lignocellulosic biomass to fermentable sugars for its conversion to ethanol. Most commercial enzyme products available today for lignocellulose hydrolysis are limited to a few fungi, amongst which *Trichoderma reesei* dominates [14]. These fungal strains are constantly being improved in terms of enzyme production using random and targeted approaches. The use of random mutagenesis for improving enzyme-producing fungi has a long and successful history. *T. reesei* RUT-C30, one of the best cellulase producers currently used globally, has been obtained after several rounds of mutations [15]. Strain improvement by a targeted approach involves the manipulation of the genome by recombinant DNA technology. Before proceeding to this approach, a comprehensive knowledge of fungi is required. The fungus *Penicillium funiculosum* NCIM1228 was screened in our laboratory for having better saccharification capability than the available commercial counterparts as its secreted enzymes could achieve 75% biomass hydrolysis within 36 h (with just ~ 0.4 mg protein per gram pre-treated biomass at 5% solid loading) [16]. Along with the CAZymes, several nonhydrolytic accessory proteins were also found to be induced in its secretome in the presence of cellulosic substrates [17]. However, a comprehensive understanding of the biomass-degrading enzyme system present in this fungus is still needed.

The cellulose-hydrolyzing enzymes are secreted by these organisms only when the plant polymers are present in the growth media. These organisms have developed sophisticated mechanisms to sense the type and composition of plant biomass and synthesize enzymes accordingly [18]. In most cases, the genes coding for hydrolytic enzymes are induced by various compounds derived from plant cell wall material or their metabolic derivatives [19]. In the presence of metabolizable sugar, such as glucose, the cells avoid excessive enzyme production by repressing these genes through carbon catabolite repression [20]. The obligatory presence of an inducer for the expression of cellulase and hemicellulase genes implies a tight co-regulation of these genes. Transcription factors (TFs) link the signal flow and the target gene expression and are essential players in signal transduction pathways [21]. The homolog of yeast carbon catabolite repressor Mig1 in fungi, also known as CreA/CRE1, has been shown to negatively regulate numerous cellulase, hemicellulase, and pectinase genes in *T. reesei* and *Aspergillus* [22, 23]. The Mig1 homolog was also identified in *P. funiculosum* NCIM1228 and was deleted from the genome to understand its function [24]. Though the Mig1-disrupted *P. funiculosum* NCIM 1228 showed higher cellulase activity, the deletion strain did not show complete de-repression of cellulase gene expression, suggesting an involvement of a complex network of TFs. In addition, *P. funiculosum* NCIM1228 has been shown to respond to different carbon substrates by secreting different sets of enzymes; thus, it is imperative to identify the TFs participating in cellulase and hemicellulase gene regulation [17, 24]. The TFs usually comprise 0.5–8% of the gene content in fungi, and despite their importance, the TF repertoires for many fungal genomes remain largely unknown [25].

In this study, we report sequencing and annotation of the *P. funiculosum* NCIM1228 genome. From the sequenced genome, the Carbohydrate-active enzyme (CAZyme) genes and Transcription Factors (TFs) were identified. Based on the protein sequence, we identified the domain structures of the annotated CAZymes and TFs. We then analyzed their expression in pre-treated wheat straw (available cellulosic biomass), Avicel (a pure form of cellulose), wheat bran (a source for hemicellulose), a composite mixture of Avicel + wheat bran (inducer of cellulolytic enzymes), and glucose (cellulolytic enzyme repressor) via transcriptome sequencing. The CAZymes were found to be differentially regulated in the presence of these carbon substrates, suggesting a regulatory mechanism for sensing and regulating these enzymes. Furthermore, the expression of the identified TFs was analyzed in different substrates and correlated with the expression of CAZymes to understand their regulatory roles. Overall, this study is a step forward in understanding the fungal cellulolytic enzyme and transcription factor network to construct a superior biocatalyst that can produce efficient and higher amounts of enzymes.

## Results

### Genome sequencing and annotation

Genome sequencing of the fungal strain *P. funiculosum* NCIM1228 was performed using the GS-FLX Titanium platform (Roche/454, Branford, USA). More than 0.9 million high-quality reads of an average read length of 438 bp were generated during sequencing. Reads that overlapped each other were assembled using the GS De Novo Assembler to form 1248 contigs, of which 888 contigs were large contigs having an average contig size of 42.6 kb and an N50 size of 303 kb (Table 1). Finally, a 37.75-Mb genome of *P. funiculosum* NCIM1228 was generated with 19-fold coverage (Table 1). The integrity of the genome assembly was evaluated to be of high quality and confidence (95.56%) based on the presence of 237 of 248 core eukaryotic genes (Table 1) [26]. In total, 10,739 protein-coding genes were predicted from the genome assembly using different gene prediction programs, such as Augustus (http://bioinf.uni-greifswald.de/augustus/), GeneMark-ES (http://exon.gatech.edu/GeneMark/), Genewise (http://www.ebi.ac.uk/Tools/psa/genewise/) and SNAP (Additional file 1). Of the coding genes, 7578 (70.5%) were annotated in the Gene Ontology (GO) database (http://geneontology.org/) and 8118 (75.5%) in the NCBI non-redundant (NR) protein sequences database (ftp://ftp.ncbi.nlm.nih.gov/blast/db/).

**Table 1:** Features of assembled genome and gene set for *P. funiculosum* NCIM1228

Features	Observations
**Genome statistics**	
Sequencing technology	Roche 454
Genome size	37.75 (Mb)
Sequencing coverage	19x
HQ reads	9,21,942
HQ bases	4038,20,878
Average read length	438
Number of contigs	1248
Number of large contigs (> 500 bp)	888
**Quality control**	
CEGMA (full length genes recovered)	95.56% (237/248)
CEGMA (partial length genes recovered)	98.39% (244/248)
**Annotation (Software used)**	
Total proteins predicted (MAKER)	10,739
Hypothetical proteins	2,621
CAZymes predicted (CAZy database)	298
TFs predicted (PFAM)	451

### Annotation of CAZymes involved in biomass degradation in *P. funiculosum* NCIM1228

**Fig. 1:**
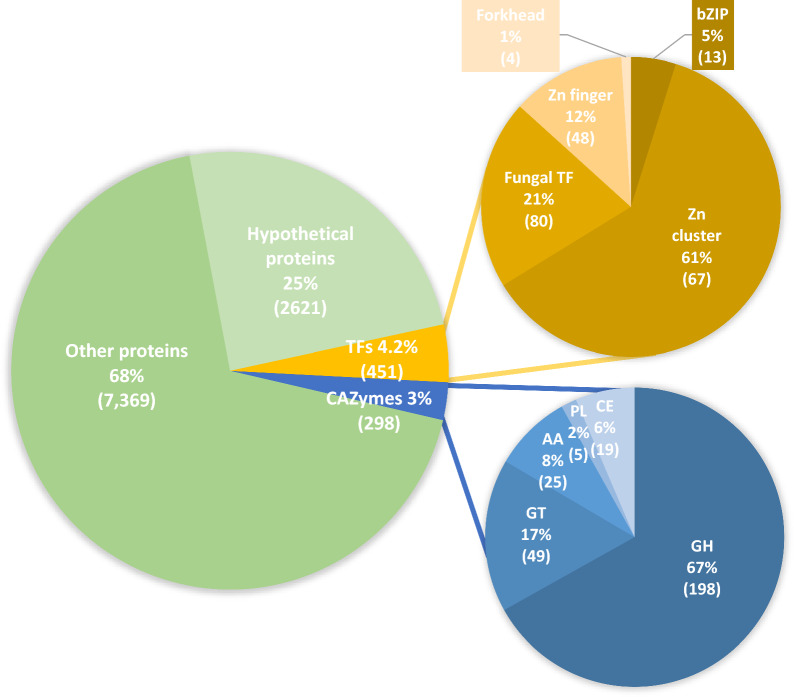
Distribution of the protein-coding genes identified in *P. funiculosum* NCIM1228 in the Carbohydrate Active Enzymes (CAZymes) and Transcription Factors (TFs) gene families. Values in parentheses represent the actual number of protein coding genes identified in each category. *GT* glycosyl transferases, *AA* auxiliary activities, *CE* carbohydrate esterases, *PL* polysaccharide lyases, *GH* glycoside hydrolases

The *P. funiculosum* NCIM1228 has already demonstrated its ability to saccharify lignocellulosic biomass [16, 17]. Several hydrolytic and nonhydrolytic accessory proteins were identified in its secretome. The genes coding for CAZymes in *P. funiculosum* NCIM1228 were identified using sequence similarity and HMM profiles to determine the complete repertoire of biomass-degrading enzymes coded by its genome. The CAZyme analysis toolkit (CAT) was used to predict modules based on sequence similarity [27]. The Carbohydrate-active enzyme ANnotation (dbCAN) database, which contains the HMM profiles for each CAZy category (http://www.cazy.org/), was used to identify CAZymes based on HMM profiles [28, 29]. The proteins identified from these methods were compared, and 298 candidate genes were annotated as CAZymes (Fig. 1, Additional file 2) using this approach. These CAZymes were divided into 52 families of glycoside hydrolases (GHs), 20 families of glycosyl transferases (GTs), 9 families of carbohydrate esterases (CEs), 8 families of auxiliary activities (AAs), and 3 families of polysaccharide lyases (PLs) genes (Fig. 1, Additional file 2). The most abundant CAZy category is GH, with 198 domains, followed by 49 domains of GT, 25 of AA, 19 of CE and 5 of PL (Additional file 2). The members of each family show similar domain architecture (Additional file 8: Figure S1). CBM1 is the most predominant CBM associated with all the members of GH families of GH6, GH7, GH10, GH11, GH45 and some members of GH5, GH18, GH54 and GH62 (Additional file 8: Figure S1). Members of CE2, CE3, and CE5 also contain the CBM1 domain. Another CBM, namely, CBM20, was identified to be associated with members of GH13 and GH15. CBM48 was found to be associated with only one member of GH13 (C0004G7.2). Some CAZymes have domains of different enzymatic functions, such as the GH28 is associated with CE8 in C0037G2.96 and with the PL3 domain in C0012G1.47 (Additional file 2, Additional file 8: Figure S1). These enzymes have been identified to have a polygalacturonase and rhamnogalacturonase function, respectively.

Based on the types of domains present in these CAZymes, the ones involved in biomass degradation were identified. A total of 88 CAZymes were predicted to be biomass-degrading enzymes, of which 32 were cellulolytic enzymes, 51 hemicellulases, and 5 pectinases (Table 2). Amongst the cellulolytic enzymes, 2 cellobiohydrolases (CBHs, EC 3.2.1.91), 8 β-endoglucanases (EGs, EC 3.2.1.4) and 16 β-glucosidases (BGLs, EC 3.2.1.21) were identified as cellulases. One lytic polysaccharide monooxygenase (LPMO) belonging to the AA9 category was also identified in the *P. funiculosum* NCIM1228 genome. Hemicellulose, a heteropolysaccharide, requires the action of several enzymes with different substrate specificities. About 51 genes coding for hemicellulases such as endoxylanase, β-xylosidase, endoglucanase, α-d-galactosidase, β-d-mannosidase, endomannanase and arabinofuranosidase were identified in *P. funiculosum* NCIM1228 genome (Table 2). Xylose hydrolyzing enzymes, such as endoxylanase and β-d-xylosidase, which can hydrolyze linear xylooligosaccharides or xylobiose, respectively, to the monomer xylose, were present in *P. funiculosum* NCIM1228. Genes coding for pectin-degrading enzymes, such as pectin esterase, pectin lyase and pectate lyase, were also identified. Two genes coding for PL1 pectin lyase, one each for PLI and PL3 family pectate lyase and one pectinesterase belonging to CE8 were identified in the genome (Table 2).

**Table 2:** Biomass degrading enzymes in *P. funiculosum* NCIM1228

Enzyme	EC Number	CAZy Family	Protein ID
1. Cellulose-degrading enzymes			
Cellobiohydrolase	3.2.1.91	GH6	C0037G1.53
		GH7	C0012G4.113
β-1,4-Endoglucanase	3.2.1.4	GH5	C0026G2.46
			C0035G2.46
			C0045G0.59
	3.2.1.75		C0007G0.69
	3.2.1.4	GH7	C0064G0.26
		GH12	C0007G4.92
		GH45	C0019G3.117
			C0041G1.26
Xyloglucan-specific β-1,4-endoglucanase		GH74	C0011G4.0
	3.2.1.151	GH12	C0042G1.114
β-1,3(4)-Endoglucanase	3.2.1.6	GH16	C0015G4.46
		GH5	C0015G2.53
			C0029G2.29
Lytic polysaccharide monooxygenases (LPMOs)		AA9	C0004G6.48
β-Glucosidase	3.2.1.21	GH1	C0006G1.88
			C0032G1.73
			C0016G3.190
			C0013G1.55
			C0113G0.42
		GH3	C0001G11.72
			C0081G0.181
			C0094G0.81
			C0006G5.25
			C0003G4.15
			C0040G0.38
			C0003G5.49
			C0018G2.2
			C0042G1.32
			C0017G2.95
			C0069G0.5
2. Hemicellulose-degrading enzymes			
α-Galactosidase	3.2.1.22	GH27	C0001G7.121
			C0012G1.39
		GH36	C0020G1.21
β-1,4-Endoxylanase	3.2.1.8	GH10	C0041G1.86
		GH11	C0015G3.0
			C0183G0.0
			C0132G0.8
			C0011G4.30
			C0095G0.55
		GH30	C0129G0.4
			C0002G4.51
β-d-Xylosidase	3.2.1.37	GH3	C0005G6.188
			C0137G0.7
			C0024G0.29
		GH43	C0013G3.69
			C0018G3.79
l-Arabinofuranosidase	3.2.1.55	GH43	C0005G0.22
			C0048G1.60
		GH51	C0052G1.192
			C0001G6.63
		GH54	C0154G0.0
			C0068G0.36
			C0074G0.133
			C0013G2.111
			C0046G0.12
		GH62	C0004G6.30
			C0003G4.112
			C0104G0.23
			C0015G3.38
β-Mannosidase	3.2.1.25	GH5	C0066G0.3
		GH2	C0017G2.58
			C0021G3.23
β-Endogalactosidase	3.2.1.89	GH53	C0094G0.18
α-Glucuronidase	3.2.1.139	GH67	C0153G0.22
Polygalacturonase	3.2.1.15	GH28	C0065G0.97
			C0138G0.1
			C0017G2.10
			C0037G2.96
			C0026G2.30
			C0061G1.52
			C0040G1.182
			C0011G2.8
Rhamnogalacturonan hydrolase	3.2.1.172	GH28	C0010G1.31
			C0012G1.47
			C0054G1.59
d-4,5-unsaturated β-Glucuronyl hydrolase	3.2.1. -	GH88	C0034G2.72
			C0059G1.60
Rhamnogalacturonan acetylesterase	3.1.1.86	CE12	C0108G0.76
Acetyl xylan esterase	3.1.1.72	CE2	C0048G1.122
		CE5	C0047G0.53
Feruloyl esterase	3.1.1.73	CE1	C0005G1.55
3. Pectin-degrading enzymes			
Pectinesterase	3.1.1.11	GH28, CE8	C0037G2.96
Pectate lyase	4.2.2.2	PL3	C0124G0.38
		PL1	C0219G0.0
Pectin lyase	4.2.2.10	PL1	C0030G0.45
			C0037G1.51

### Annotation of transcription factors in *P. funiculosum* NCIM1228

Biomass-degrading enzymes in a cell are expressed only under stringent conditions. Regulation of gene expression for these enzymes in a cell is primarily coordinated by the transcription factors (TFs). To identify the TFs involved in regulating enzyme production, we require an exhaustive list of genes coding for TFs in the *P. funiculosum* NCIM1228 genome. All the genes coding for the TF families reported by Shelest E. 2008 were retrieved from the *P. funiculosum* NCIM1228 genome [21]. After manually inspecting the PFAM families and their functional domains, genes coding for proteins other than transcription factors were discarded. We found that the genome of *P. funiculosum *NCIM 1228 codes for 451 TFs, accounting for 4.2% of its estimated total number of genes (Fig. 1, Additional file 3). Categorized by the DNA-binding domain, the TFs fall into a handful of classes as shown in Fig. 1 and listed in Additional file 3. Zinc-binding proteins form one of the most prominent families of transcriptional regulators in eukaryotes [30]. These are categorized based on their structure as either Zn-finger Class-I, Class-II or the Zn-cluster proteins. The Zn-finger Class-I and II proteins have been identified as TF in all the eukaryotes, while Zn-cluster proteins have been identified exclusively in fungi [30]. In *P. funiculosum* NCIM1228, Zn-binding proteins form the largest family of transcription regulators. Of this, the Zn cluster domain (pfam00172) is the most abundant (240 proteins), followed by Zn-finger class 1 (39 proteins) and Zinc finger class 2, including the GATA factors (7 proteins) (Fig. 1). Following the Zn-binding proteins, the second-largest TF class is the ‘fungal-specific transcription factor domain’ (pfam04082) containing proteins (222 proteins). This domain is present either with the Zn-finger domain (142 proteins), with the MFS domain (2 proteins) or without any other domain (78 proteins) (Additional file 8: Figure S2). Another domain, ‘fungal-specific transcription factor domain_2’ (pfam11951) was present in 42 proteins. This domain is primarily present with the Zn-cluster domain (pfam00172).

We carried out a homology search of known TFs involved in regulating lignocellulolytic genes in filamentous fungi against the translated proteins in the *P. funiculosum* NCIM1228 genome (Additional file 8: Table S1). Homologs of most known TFs were identified in *P. funiculosum* NCIM1228, including the cellulase transcription activator ClrB, the starch degradation regulator AmyR and the xylan degradation regulator XlnR (Table 3). There were several other related proteins, including ACEII, Xpp1, Clr-1 and BglR, whose homologs could not be identified in *P. funiculosum* NCIM1228.

**Table 3:** Homologs of known transcription factors involved in the regulation of biomass-degrading enzyme genes in *P. funiculosum* NCIM 1228

S. no	Protein	Species	Accession No	Protein ID
1	AceA	*P. oxalicum*	EPS27047.1	Not found
2	AceII	*T. reesei*	AAK69383.1	Not found
3	AreA	*A. nidulans*	CAA36731	C0067G0.23
4	AraR	*A. niger*	A2QJX5.1	C0006G0.30
5	AmyR	*P. oxalicum*	EPS29018.1	C0012G2.24
6	BglR	*T. reesei*	EGR44729.1	Not found
7	BrlA	*P. oxalicum*	EPS25156.1	C0016G0.20
8	Clr-1	*N. crassa*	ESA42840	Not found
9	ClrB	*P. oxalicum*	EPS31045.1	C0021G3.75
10	ClrC	*P. oxalicum*	EPS34061.1	C0127G0.8
11	Hap2	*P. oxalicum*	EPS31428.1	C0020G2.1
12	Hap3	*P. oxalicum*	EPS27888.1	C0029G1.88
13	Hap5	*P. oxalicum*	EPS26080.1	C0099G0.143
14	PacC	*N. crassa*	Q7RVQ8.2	C0040G1.97
15	FlbC	*P. oxalicum*	EPS33410.1	C0019G3.93
16	Rca1	*N. crassa*	XP_961398.1	C0037G1.47
17	Vib1	*N. crassa*	XP_011394570.1	C0088G0.11
18	XlnR	*P. oxalicum*	EPS32714.1	C0081G0.41

### Transcriptomic profiles of *P. funiculosum* NCIM1228 in response to different carbon substrates

Secretome analysis of *P. funiculosum* NCIM1228 has shown high cellulolytic activity when it was cultivated in the presence of cellulose and hemicellulose, and its secretome pattern has shown variations in the enzymes expressed with different carbon substrates [16, 17]. Our genome analysis predicted that *P. funiculosum* NCIM1228 encodes for a vast repertoire of CAZymes and TFs. To identify the CAZymes expressed in different carbon substrates and the TFs regulating their expression, we sequenced the *P. funiculosum* NCIM1228 transcriptome cultivated in five different carbon substrates (i) Avicel, (ii) wheat bran, (iii) Avicel + wheat bran, (iv) pretreated wheat straw and (v) glucose. Glucose represses cellulase/hemicellulase expression by activating carbon catabolite repression. Wheat bran is heterogeneous in nature and composed of starch and non-starch polysaccharides (NSP). Arabinoxylan is a major component of NSP, and thus wheat bran is considered to be a good inducer of hemicellulases [31, 32]. Avicel is a pure, homogeneous micro-crystalline cellulose and a known inducer of core cellulases [12]. Ammonium hydroxide pre-treated wheat straw used in our experiment is considered as a potential feedstock for second-generation biofuels, and is heterogeneous but majorly contains crystalline cellulose. A composite mixture of Avicel with wheat bran in equal proportion served as a model substrate for induction of cellulases and hemicellulases as reported in our previous study [16].

To identify the appropriate timepoint for transcriptome sequencing, we estimated the endoglucanase, xylanase and β-glucosidase activity of the *P. funiculosum* NCIM1228 supernatant each day up to five days (Additional file 8: Figure S3). A rise in endoglucanase and xylanase activity was observed till day 3, while β-glucosidase activity increased till day 4, after which the activities almost saturated. Since transcriptional changes would precede metabolic changes, we selected 2.5 day (60 h) as the timepoint for RNA isolation and transcriptome sequencing. Since the assays used for estimating the cellulolytic activities are based on the estimation of the reducing sugar produced in the enzymatic reaction, estimation was not possible for cells cultivated in glucose. For comparison with cells cultivated in glucose, we estimated the total protein concentration of the supernatant. At day 2, the protein concentration was maximum so we selected 1.5 days (36 h) as the timepoint because growth was faster in glucose as compared to the complex polymeric substrates.

Ten barcoded libraries (5 samples in duplicates) were sequenced using the Illumina Hiseq 2000 System, generating approximately 663 million reads (357 million reads and 306 million reads for both the biological replicates, respectively) (Additional file 8: Table S2). High-quality reads of each condition were filtered via NGSQC Toolkit, and only those passing the filter were used for further analysis (Additional file 8: Table S2). The high-quality reads for each replicate were mapped separately on the *P. funiculosum* NCIM1228 genome sequence using TopHat software. The summary of sequence data generated, filtered reads and reads mapped on the genome is given in Additional file 8: Table S2. There was a high correlation (Pearson correlation, *R* ≥ 0.95) between the two replicates of each condition used in the transcriptional analysis (Additional file 8: Figure S4A). After sample normalization, boxplots were constructed to determine if the conditions were comparable, and the results are presented in Additional file 8: Figure S4B. The mapped read files were used for reference-guided assembly via the Cufflinks–Cuffmerge pipeline. Differential gene expression was analyzed using the Cuffdiff platform. The abundance of the transcripts for each replicate was measured in the number of fragments per kilobase of exon per million fragments mapped or the FPKM.

### Expression profile of *P. funiculosum* NCIM1228 CAZymes in response to different carbon substrates

**Fig. 2:**
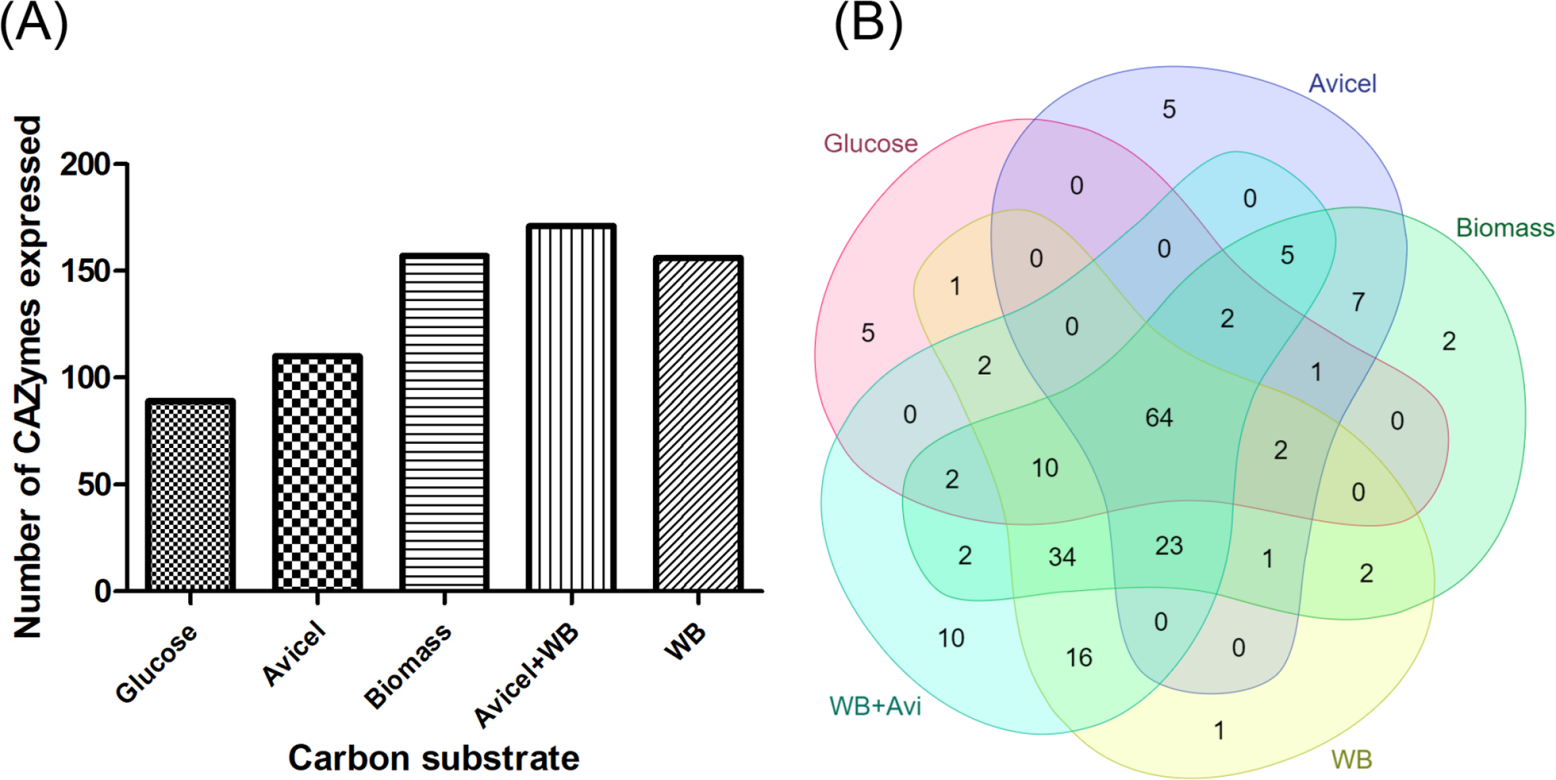
*P. funiculosum* NCIM1228 CAZyme expression in response to different carbon substrates. **A** Bar graph shows the total number of CAZymes induced in the presence of different carbon substrate used for *P. funiculosum* NCIM1228 cultivation. The CAZymes having significant expression (> 1 FPKM) and expressed in duplicate samples were identified for all the carbon substrates. **B** Venn diagram showing the distribution of the CAZymes expressed across the carbon substrates

The ability of the carbon substrates to induce the various genes coding for CAZymes in *P. funiculosum* NCIM1228 was studied. The CAZymes having expression greater than 1 FPKM in both the duplicate samples were considered expressed and used for further analysis. The results found that the least number of CAZymes were expressed in glucose (89), followed by Avicel (110). A maximum number of CAZymes were expressed in WB + Avi (171), and similar number of CAZymes were expressed in WB (156) and Biomass (157) (Fig. 2A). Upon further analyzing the distribution of these induced CAZymes, it was found that there were 10 unique CAZymes that were expressed in WB + Avi (Fig. 2B, Additional file 4), which included—FAD-oxidase, polygalacturonase, rhamnogalacturonan α-l-rhamnopyranohydrolase, α-glucosidase and β-galactosidase. In Avicel, AA enzymes such as glucose oxidase and NADH-quinone oxidoreductase were expressed. Biomass induced an inulinase and an arabinofuranosidase. There were 64 CAZymes that were expressed in all the five carbon substrates.

**Fig. 3:**
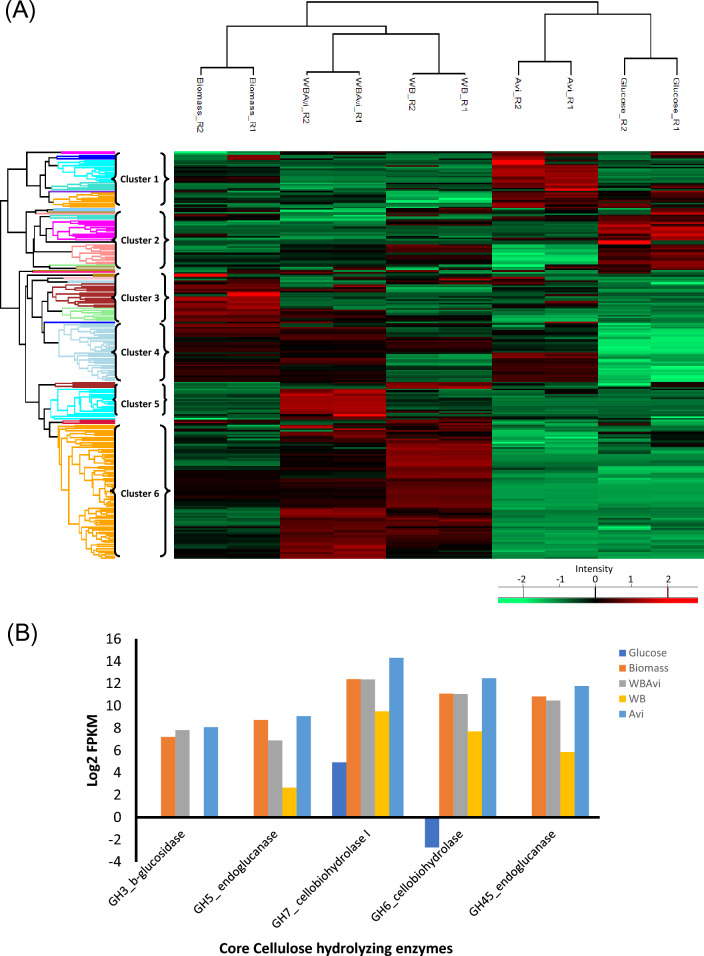
Expression profile of *P. funiculosum* NCIM1228 genes coding for CAZymes cultivated on different carbon substrates. **A** The expression values of the duplicate samples for each carbon substrate were used to plot the heat map. The data for each gene is represented as rows (for gene IDs from top to bottom see Additional file 5), and the carbon substrates used for cultivation are shown as columns. The heat map was divided into 7 clusters according to similarities in expression profiles. **B** Bar graph represents the expression of key cellulose hydrolyzing enzymes in the five carbon substrates. The contig ID of the enzymes are as follows - GH3_b glucosidase: C0069G0.5, GH5_endoglucanase: C0026G2.46, GH7_cellobiohydrolase I: C0012G4.113, GH6_cellobiohydrolase: C0037G1.53, GH45_endoglucanase: C0019G3.117

PCA analysis suggested variations in different carbon substrates but not in the duplicate samples (Additional file 8: Figure S5A). The FPKM values of the CAZymes (in duplicates) were clustered to generate a heat map to study the differences in the expression patterns (Fig. 3). A considerable difference was observed in the expression pattern of all five carbon substrates. The transcriptomes of homogeneous carbohydrates—glucose and Avicel, were found to be neighbours, whereas transcriptomes of heterogeneous substrates wheat bran and wheat bran + Avicel were found to be relatively close. The transcriptome of biomass formed a separate clade but was closer to that of wheat bran + Avicel.

The heat map was divided into six clusters based on the expression pattern. Enzymes displaying high expression in Avicel and low expression in other carbon substrates were represented in Cluster 1, consisting of 33 genes (Additional file 5). Of these genes, two critical genes coding for secretory proteins of functional relevance were identified. A GH72 glucanosyltransferase (GH72_a) and an AA9 LPMO (AA9_a) showed high expression in Avicel and low expression in other carbon substrates. Both showed almost negligible expression in glucose. An earlier report by Ogunmolu et al. also suggests that AA9 activity was found to be maximum in the case of Avicel [15]. Some of the other hydrolyzing enzymes, such as GH17 GPI-anchored cell wall β-endoglucanase (GH17_a), GH5 exoglucanase (GH5_e), GH13 glycogen branching enzyme (GH13_c), GH31 α-glucosidase (GH31_a) and GH63 α-glucosidase (GH63_b) were expressed in all the carbon substrates at an almost similar level. Of these, the enzymes β-endoglucanase (GH17_a), α-glucosidase (GH31_a) and α-glucosidase (GH63_b) were secretory proteins suggesting their action on the carbon substrates present in the cultivation media, while the exoglucanase (GH5_e) did not contain a signal peptide and might be involved in cell wall remodelling. Three chitinases—GH18_c, GH18_d, and GH18_k were identified in this cluster but were only marginally expressed. Only the chitinase GH18_k was a secretory protein, and the other two GH18_c, GH18_d, without the secretary signal, might be active against chitin components for cell wall remodelling.

Cluster 2 contained genes that were induced majorly in response to glucose. AA2 (AA2_b), a bifunctional catalase-peroxidase, showed higher expression in glucose than the other substrates. Glycosyl hydrolases such as GH31 α-glucosidase (GH31_b) showed high expression in glucose, whereas GH32 inulinase (GH32_d) and GH13 α-amylase (GH13_a) displayed high expression in both WB and glucose. The rest of the glycosyl hydrolases, such as GH31 α-glucosidase (GH31_h), GH1 β-Glucosidase (GH1_d), GH15 glucoamylase (GH15_a, GH15_b), GH13 α-amylase (GH13_b, GH13_f) showed highest expression in glucose followed by other substrates and least in the crystalline substrate Avicel. All these GHs contained a secretory peptide suggesting that they are secreted from the cell.

Cluster 3 included enzymes that were majorly induced in response to Biomass. Biomass being a heterogenous polysaccharide induced a mixture of enzymes capable of cleaving the them, such as GH43 xylosidase (GH43_a), GH62 arabinofuranosidases (GH62_d) and GH32 inulinase (GH32_e). A GH5 endoglucanase and a PL20 lyase were also induced. Some of the chitinases such as GH18_f and GH18_j were found in this cluster. GH18_f was almost equivalently expressed in all the conditions indicating its involvement in cell wall remodelling. Whereas the other chitinase, GH18_j, which contained a signal peptide was only expressed in biomass and WB + Avi suggestive of its action on the carbon substrate present in the cultivation media.

Cluster 4 exhibited enzymes induced in response to all the cellulosic substrates, such as Biomass, WB + Avicel, and Avicel, and they were expressed in least quantity in glucose. The majority of the cellulose hydrolyzing enzymes such as GH6 and GH7 cellobiohydrolase (GH6_a, GH7_a, GH7_b) and GH5 endoglucanase (GH5_h) and GH76 α-1,6-mannanase (GH76_b) were found to be highly expressed in Avicel, followed by other substrates, such as biomass and the mixture of WB + Avicel. All these proteins had a secretory peptide associated with them suggesting induction for action on specific substrates. β-Glucosidases (GH3_a, GH3_c, GH3_i, GH3_l) were also highly expressed in this cluster. Carbohydrate Esterases (CE), which are an important set of enzymes for the hydrolysis of cellulolytic and hemicellulolytic substrates, were found to be induced in this cluster (CE2_a, CE1_b, CE5_a, CE3_b, CE3_a, and CE10_e). Hemicellulolytic enzymes such as GH10 and GH11 endoxylanase, GH5 mannosidase, GH43 xylosidase, GH62 arabinofuranosidase and GH76 α-1,6-mannanase (GH76_b) also appeared in this cluster and showed relatively higher expression in WB, Biomass and WB + Avicel (Fig. 3, Additional file 5).

Cluster 5 comprises enzymes induced majorly in response to WB + Avicel, such as α-mannosidase, α-glucosidase, β-glucuronidase, α-rhamnopyranohydrolase, and polygalacturonase. Cluster 6 exhibited enzymes induced in response to WB-containing substrates such as WB itself and WB + Avicel. The majority of the enzymes identified were hemicellulases, such as GH62 arabinofuranosidase (GH62_c), GH11 β-xylanase (GH11_c, GH11_g, GH11_e), GH13 (GH13_h) and GH31 α-glucosidase (GH31_c), GH51 (GH51_b) and GH54 α-arabinosidase (GH54_e), GH2 β-mannosidase (GH2_c), GH30 β-1,6-glucanase (GH30_a, GH30_b, GH30_c) and GH71 α-glucanase (GH71_c).

This analysis gives us an insight into the CAZymes induced by different carbon substrates. The data indicates that different carbon substrates exhibit divergent gene expression profiles, and they most often induce enzymes needed for their metabolism. In this way, the organism conserves its energy by producing only the necessary enzymes for its growth and survival. Altogether, this study indicates a complex regulatory mechanism that controls the expression of these enzymes.

### Expression profile of *P. funiculosum* NCIM1228 transcription factors (TFs) in response to different carbon substrates

**Fig. 4:**
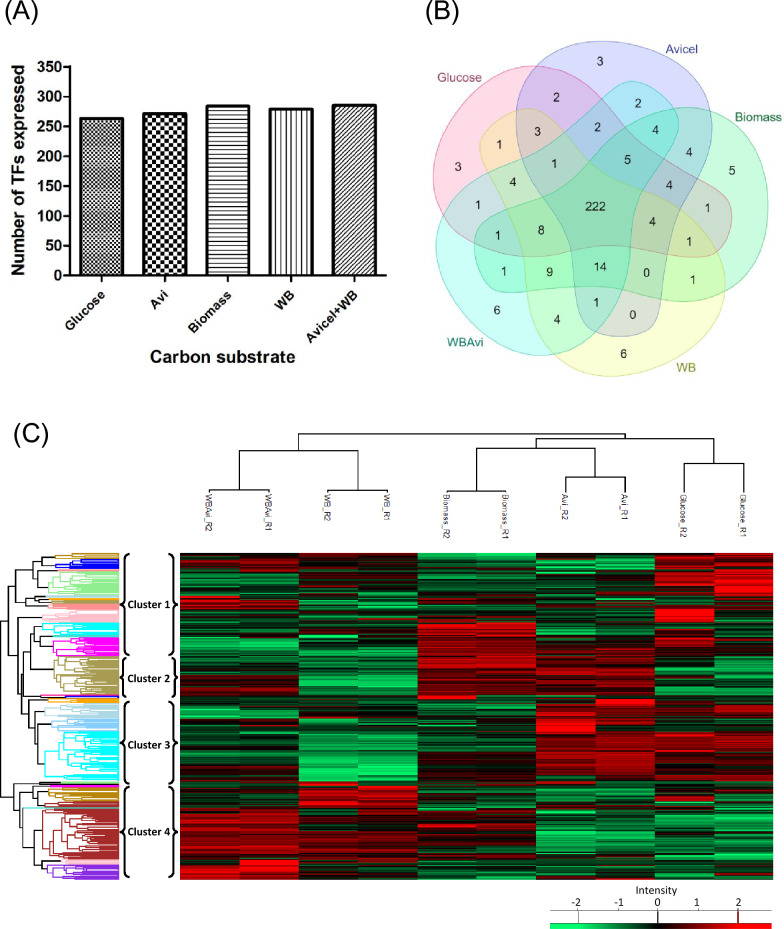
*P. funiculosum* NCIM 1228 TFs expression in response to different carbon substrates. **A** Bar graph shows the total number of TFs induced in the presence of different carbon substrate used for *P. funiculosum* cultivation. The TFs having significant expression (> 1 FPKM) and expressed in duplicate samples were identified for all the carbon substrates. **B** Venn diagram showing the distribution of the TFs expressed across the carbon substrates. **C** Expression profiles of *P. funiculosum* NCIM 1228 genes coding for TF cultivated on different carbon substrates. The expression values of the duplicate samples for each carbon substrate were used to plot the heat map. The data for each gene is represented as rows (for gene IDs from top to bottom see Additional file 7), and the carbon substrates used for cultivation are shown as columns. The heat map was divided into 4 clusters according to similarities in expression profiles

To identify the TFs regulating the CAZymes, we investigated the expression of the TFs identified in *P. funiculosum* NCIM 1228 in all the carbon substrates. The expression of TFs was evaluated based on the FPKM values of their transcripts obtained in the different carbon substrates. Only the TFs having an expression of more than 1 FPKM in both duplicate samples were considered expressed and used for analysis. Upon analyzing the results, we found that the least number of TFs were expressed in glucose (263), followed by Avicel (271) and WB (279) and a similar number of TFs were expressed in WB + Avicel (285) and biomass (284) (Fig. 4A). Unique TFs expressed in each carbon substrate were identified by generating a Venn diagram (Fig. 4B, Additional file 6). A maximum of 6 unique TFs were expressed in WB + Avi and WB (Fig. 4B, Additional file 6). Around 222 TFs were identified to be expressed in all five carbon substrates indicating the involvement of a large set of TFs in all five carbon substrates (Additional file 6).

PCA analysis suggested variations in different carbon substrates but not in their replicates (Additional file 8: Figure S5B). The FPKM values of the TF transcripts were clustered to generate a heat map to study the differences in the expression patterns (Fig. 4C). A considerable difference in the expression pattern was observed in all five carbon substrates. The transcriptomes of Avicel and Biomass were found to be grouped together, whereas transcriptomes of wheat bran and Avicel + wheat bran were grouped together. Glucose formed a separate clade, but was close to Avicel and Biomass group. Based on the expression pattern, the heat map was divided into four clusters (Additional file 7). Most members of cluster 1 were expressed in all the substrates, including glucose. Since the TFs belonging to this cluster were not induced in a specific carbon substrate, this cluster was not relevant to identify TFs related to the hydrolysis of cellulose or hemicellulose in the biomass. Only one of its members, a bZIP TF (bZIP_2_f; C0133G0.11), had high expression in glucose than the other substrates, so it might be a repressor (Additional file 8: Figure S2).

Cluster 2 caught our attention as TFs belonging to this cluster were expressed in all the polymeric substrates and had low expression in glucose. These TFs might regulate the synthesis of biomass hydrolyzing enzymes. Amongst them was a bZIP transcription factor (bZIP_1_c; C0016G2.76) which showed a significantly high expression in all the carbon substrates except glucose. Further analysis suggested that this TF was homologous to *Talaromyces marneffei* Atf21. Another TF of the Zn_clus family (Zn_clus_ay; C0108G0.26) showed a similar expression pattern and significantly differed in expression in Avicel and glucose. It was homologous to the C6 transcription factor (Fcr1) of *T. marneffei* ATCC 18224. TF FT2_Zn_clus_g (C0006G5.22) had high expression in Avi and WB + Avi and low in glucose. TF bZIP_1_a (C0004G0.31) had high expression in all the carbon substrates except glucose and showed a significant difference in WB + Avi with respect to glucose.

Cluster 3 contained TFs having high expression in Avicel and glucose and also expressed in WB + Avi, WB, and biomass. This suggested that TFs present in this cluster are active in all the conditions and might be involved in regulating the basic cell cycle machinery. Homologs of *A. nidulans* AreA, and *N. crassa* PacC were identified in this cluster. The expression levels of all these TFs were almost equivalent in all the conditions, except *N. crassa* PacC homolog which showed low expression in WB than the other substrates.

The cluster 4 contained TFs expressed in WB + Avi, WB, and biomass, and hence these TFs might be involved in regulating hemicellulose expression. *P. oxalicum* Clr-B homolog was identified in cluster 4, but its expression was considerably low in all the carbon substrates. A fungal-specific TF C0099G0.5 was highly expressed in WB and WB + Avi but not in other substrates indicating its role in regulating the expression of enzymes involved in WB hydrolysis. Another TF C0057G1.124 having a fungal-specific transcription factor domain showed high expression in WB followed by WB + Avi and biomass. Its homolog could not be identified indicating that it has not been previously characterized.

**Fig. 5:**
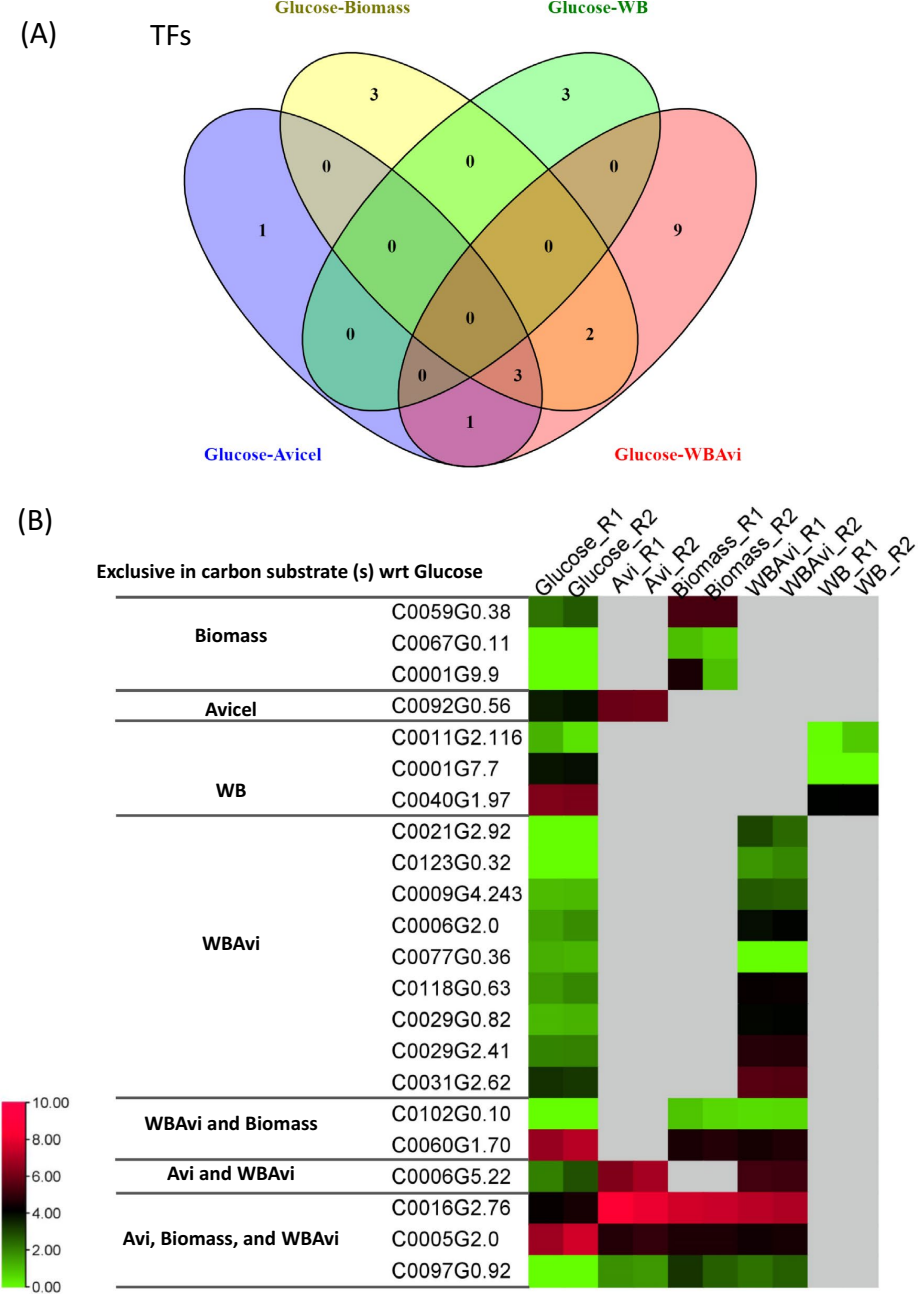
Differentially expressed TFs identified in *P. funiculosum* NCIM 1228. **A** Venn diagram summary of differentially expressed genes coding for TFs with respect to glucose having a log2 fold change ≥ twofold, at a *P* value ≤ 0.01. **B** Expression profile of differentially expressed genes of *P. funiculosum* NCIM 1228 coding for TFs cultivated on different carbon substrates. Expression values of TFs which are not significant with respect to glucose are represented by grey boxes

Furthermore, we identified the genes coding for TFs that were significantly expressed (*P* < 0.05) with respect to glucose (Fig. 5A, Additional file 8: Table S3). The TFs having a significant change in the expression with respect to glucose (*P* value) in both the duplicates were compared and analyzed (Fig. 5A, Additional file 8: Table S3). The Venn diagram represents three TFs which were differentially regulated in Avicel, biomass and WB + Avi compared to glucose (Fig. 5A). Of these three TFs, the TF C0016G2.76 exhibited high expression in all these three carbon substrates but had significantly less expression in glucose (Fig. 5B). This suggested that this TF could regulate the enzymes hydrolyzing biomass. Another TF, C0006G5.22, had higher expression in Avicel and WB + Avi than glucose. A fungal TF C0092G0.56 and a Zn cluster TF C0059G0.38 had high expression in Avicel and Biomass, respectively. These TFs might be playing a role in regulating biomass hydrolysis. The *N. crassa* PacC homolog C0040G1.97 had significantly higher expression in glucose than WB suggesting its role in regulating WB hydrolyzing enzymes.

In addition, we validated the expression pattern of some of these TFs using RT-qPCR. The expression levels of these TFs were compared on glucose and Avicel (Additional file 8: Figure S6). Most of the TFs showed similar expression levels in both Real-Time PCR and Illumina RNA-Seq expression profile (Additional file 8: Table S4), validating the Illumina RNA-Seq expression profile data.

## Discussion

Filamentous fungi such as *T. reesei*,* Aspergillus niger*, and *Talaromyces cellulolyticus* can secrete a plethora of biomass hydrolyzing enzymes [33–35]. The secreted consortia of hydrolytic enzymes include CAZymes that are highly efficient in the degradation of complex biomass into monomeric sugars. *T. reesei* is one of the leading commercial sources of cellulolytic enzymes, and several of its mutant strains such as QM6a, QM9414 MCG77, and RUT C30 have been developed for industrial use [36]. These strains are being used commercially for the deconstruction of agriculture and forestry residues, woody biomass, and dead trees for the production of bioethanol [37].

One of the fungal strains, *P. funiculosum *NCIM1228, has great potential to produce enzymes relevant to the efficient degradation of lignocellulosic biomass [16]. It secretes a repertoire of inducible hydrolytic CAZymes and non-hydrolytic accessory proteins, which synergistically mediate the action of biomass hydrolysis [17]. Its secretome possesses β-glucosidase activity comparable to the mutant strain of the RUT-C30/commercial cellulase cocktail, indicating effective enzymes [16]. Though a few CAZymes were identified in the *P. funiculosum* NCIM1228 secretome, the complete set of CAZymes coded in *P. funiculosum* NCIM1228 needed to be annotated. This prompted us to investigate the list of CAZymes coded in its genome. We first sequenced, assembled, and analyzed the genome of *P. funiculosum* NCIM1228. Its total assembled genome size was estimated to be 37.75 Mb, which was greater than the genome size of other filamentous fungi, including some species of the genus *Penicillium* (Additional file 8: Table S5) [38]. Its genome size and properties were found to be closer to that of *Talaromyces pinophilus* (Additional file 8: Table S5) [39, 40]. Around 10,739 protein-coding genes were identified in its genome, which was in the similar range to that of other species of the genus *Talaromyces* and *Penicillium* that have been reported to be cellulase producers (Additional file 8: Table S5). Genome analysis of *P. funiculosum* NCIM1228 led to the annotation of 298 CAZymes, which helped us to identify significant biomass hydrolyzing enzymes (Fig. 1). A total of 16 β-glucosidases were identified in the *P. funiculosum* NCIM1228 genome, that were higher than the 11 β-glucosidases identified in the *T. reesei* genome [41]. In addition, the considerable high β-glucosidase activity identified in *P. funiculosum* NCIM1228 secretome validated their presence in the genome [16].

Several studies have pointed out that fungi have sophisticated mechanisms to ensure the synthesis of enzymes only in the presence of complex carbohydrate plant polymers. They can sense the type and composition of plant biomass and synthesize enzymes accordingly. The synthesis of these enzymes is induced by low molecular weight carbohydrates and repressed by glucose or other readily metabolized sugars. We, therefore, sequenced the *P. funiculosum* NCIM1228 transcriptome to study the expression pattern of the CAZymes in different carbon substrates, such as Avicel, wheat bran, Avicel + wheat bran, pre-treated wheat straw, and in glucose that served as a control. Avicel + wheat bran induced a maximum number of CAZymes and some unique CAZymes that were not induced by other substrates (Fig. 2). A previous report by Ogunmolu et al. has also suggested that in a composite mix of Avicel and wheat bran, *P. funiculosum* NCIM1228 secretes the maximum number of hydrolyzing enzymes [16]. Since a variation in the pattern of CAZyme expression was observed (Fig. 3), we speculated that there must be key mediators in controlling the gene expression.

Transcription factors (TFs) orchestrate gene expression in a cell and determines the functionality of the cell. They are essential in the signal transduction pathways, the last link between signal flow and target gene expression [21]. Identifying TFs involved in the transcriptional regulation of cellulase and hemicellulase gene expression has been a significant effort in the past 10 years. Numerous cellulase, hemicellulase, and pectinase genes are regulated by Cys2His2-type transcription factor CreA/CRE1 proteins in *T. reesei* and *Aspergillus* species [22, 23]. Its homolog Mig1 has already been identified and studied in *P. funiculosum* NCIM1228 [24]. Disruption of Mig1 increased the production of cellulolytic enzymes—cellobiohydrolase and endoglucanase to a considerable extent [24]. However, to further identify the TFs regulating the CAZyme production, it was essential to identify all the TFs coded in its genome. A total of 451 TFs were identified and were almost similar in number to the 381 TFs identified in another ascomycete *Ascochyta rabiei*, validating the specific identification of TFs [25]. The maximum number of *P. funiculosum* NCIM1228 TFs belonged to the fungal-specific TF category, which is the case with most other fungi [21].

A breakthrough has been achieved for *T. reesei* and *A. nidulans*, where several positive and negative-acting genes were identified. The general cellulase and hemicellulase gene expression activators have been cloned and characterized from *Aspergillus* (XlnR) and *T. reesei* (Xyr1) [32]. The homologs of most of the previously identified TFs involved in regulating cellulase gene expression, such as *P. oxalicum* Clr-B, Clr-C, *N. crassa* PacC, *A. nidulans* AreA and *A. niger* AraR, were identified in the *P. funiculosum* NCIM1228 genome (Table 3). Transcriptome analysis of these TFs exhibited expression in most of the carbon substrates, but not a significant fold-change was observed with respect to glucose. The *A. nidulans* AreA homolog C0067G0.23 and *P. oxalicum* AmyR homolog C0012G2.24 showed almost an equivalent expression level in all the carbon substrates. Only the PacC homolog C0040G1.97 had a significant expression in glucose compared to WB. This suggested that though these homologs might be involved in regulating the cellulase gene expression in *P. funiculosum* NCIM1228, some other key TFs must be regulating the mechanism.

The expression of the TFs identified in its genome was visualized using a heat map (Fig. 4). The ones having high expression in all the carbon substrates other than glucose were identified here (Fig. 4). These TFs differed from the previously known TFs involved in cellulolytic enzyme production. The TFs having significantly higher expression than glucose were identified (Fig. 5). One of the TFs identified here was C0016G2.76, which had significantly higher expression in the cellulosic substrates Avicel, biomass and WB + Avi than glucose. This TF was determined to be a *T. marneffei* ATF21 homolog (Additional file 8: Figure S7). It is activated by various stresses and controls many processes, such as development, apoptosis and inflammation. It is strongly expressed under sorbitol stress conditions and might play a role in regulating the osmotic stress response [42, 43]. It has also been seen that Atf21 is indispensable for the adaptive response to several stresses, such as nitrogen starvation and for meiotic events, including nuclear divisions [44]. It forms a homo/heterodimer through its leucine–zipper domain, which binds to cyclic AMP response element (CRE: TGACGTCA) sequences, often located within promoter regions. Interestingly, ATF/CREB proteins and their characteristics are widely conserved in many species, from yeasts to humans, suggesting they play critical roles in cellular functions.

Another TF, C0006G5.22, had significantly higher expression in Avi and WB + Avi than glucose. It is a NosA homolog, which activates the fruiting body development (Additional file 8: Figure S7) [45]. NosA was transcriptionally upregulated upon starvation and during late asexual development. The latter condition is also characterized by starvation because the conidiophores are growing into the air and might be nutrient-limited. NosA appeared to regulate the expression of the high-affinity hexose transporter, HxtA and the catalase-peroxidase, CpeA, which usually are induced upon nutrient limitation, suggesting a positive role of NosA during starvation [46, 47]. The TF C0108G0.26, an Fcr1 homolog, had higher expression in Avicel and Biomass than glucose. A fungal-specific TF C0092G0.56 and a Zn cluster TF C0059G0.38 were also identified that remain uncharacterized.

Our observation suggested that the mechanisms of CAZyme regulation followed by *P. funiculosum* NCIM1228 differ from those reported for other known fungi. Upon analysing previously characterized homologs of these TFs, it was found that some are involved in fruiting body development, protein secretion and stress response. This suggested that the cells modify the basic cell cycle machinery to overcome the stress. This work thus identified TFs which might be involved in regulating the synthesis of biomass hydrolyzing enzymes.

## Materials and methods

### Microorganism, cultivation condition and genomic DNA extraction

*Penicillium funiculosum* NCIM1228 [16] was maintained and cultivated on potato dextrose agar plates (PDA; Difco Laboratories, pH 5.2–5.5) at 30 °C for 7–10 days [48]. Mycelium was cultivated in potato dextrose broth (PDB; Difco Laboratories, pH 5.2–5.5) at 30 °C for 3 days. The mycelium was filtered using Miracloth (Calbiochem, Merck, Germany) and the filtered mycelia was crushed in liquid nitrogen. Genomic DNA was isolated using YeaStar™ Genomic DNA Kit (Zymo Research, USA) according to the manufacturer’s protocol.

### Genome sequencing, assembly, and annotation

The *P. funiculosum* NCIM1228 genomic DNA was sequenced using the GS-FLX Titanium platform (Roche/454, Branford, USA). An FLX shotgun library and an 8-kb paired-end library were prepared for sequencing using the GS-FLX Titanium platform (Roche454, Branford, USA). Quality-filtered sequences from whole genome shotgun sequencing were assembled using the GS De Novo Assembler (NEWBLER Version 2.6; Roche). Reads that overlapped each other were joined into contigs. Further genome analysis was performed using these contigs. Protein-coding genes in the *P. funiculosum* genome were annotated using the MAKER annotation pipeline [49]. MAKER predicts proteins based on homology with protein-coding sequences of other species and with the consensus of the ab initio gene prediction algorithms SNAP, AUGUSTUS, and GeneMark. For protein prediction, the NCBI NR database (update 05, 2015) and fungal ESTs were used. All predicted proteins were annotated using BLASTP version 2.2.28 + search against the NCBI NR database (with the Swiss-Prot and TrEMBL databases) to assign general protein function profiles using a cutoff *E* value ≤ 1e−5. InterProScan (http://www.ebi.ac.uk/interpro/) and Gene Ontology (GO) (http://geneontology.org/) were also used to annotate the predicted proteome.

### Identification of carbohydrate-active enzyme (CAZymes) and transcription factors (TFs) in *P. funiculosum* NCIM 1228

CAZymes were identified using the CAZyme analysis toolkit (CAT) [27] as well as predicted using the database of Carbohydrate-active enzyme ANnotation (dbCAN) [29]. CAT predicts modules based on sequence similarity as well as on the links it has generated between Protein families (Pfam) and CAZy families [27]. Carbohydrate-active enzyme ANnotation (dbCAN) has a set of HMM profiles for each CAZy class based on which it predicts the modules [29]. CAT (v2.0) was used at default parameters for annotation of CAZymes, whereas dbCAN (release 3.0) was used at an *E* value cutoff of *E* value < 1e−5 if the alignment was greater than 80aa, otherwise at an *E* value < 1e−3; with coverage greater than 0.3 to predict various CAZymes. CAZymes identified were compared, and only those predicted by both CAT and dbCAN were used for further analysis. Information on the domain architectures of the proteins predicted to contain multiple domains was obtained from HMM scan data of dbCAN.

### Cultivation conditions and enzymatic activity

Conidial suspensions of *P. funiculosum* NCIM 1228 were prepared by growing it on PDA for 7 days at 30 °C. Spores were collected from lawns of fungi culture in sterile distilled water, filtered through a glass wool plug to remove hyphal fragments and counted on a hemocytometer. Conidia at 10^6^ spores mL^−1^ were inoculated in a base medium—KH_2_PO_4_ 2.0 g L^−1^; (NH_4_)_2_SO_4_ 1.4 g L^−1^; Urea 0.3 g L^−1^; MgSO_4_⋅7H_2_O 0.3 g L^−1^; FeSO_4_⋅7H_2_O 5.0 mg L^−1^; MnSO_4_⋅H_2_O 1.6 mg L^−1^ and ZnSO_4_⋅7H_2_O 1.4 mg L^−1^ [50], containing 2% carbon sources—glucose, Avicel, wheat bran, ammonium hydroxide pretreated wheat straw (named biomass in the text) and a composite combination of Avicel + wheat bran, respectively. The basal medium containing glucose served as a control. Submerged culture experiments were carried out in 100 mL shake flasks containing 20 mL cultures in duplicates. The supernatant from the culture was withdrawn each day for 5 days to test the cellulolytic and hemicellulolytic activity. The endoglucanase (β-1,4-endoglucanase), xylanase (β-1,4-endoxylanase), and cellobiohydrolase (exo-1,4-β-glucanases) activities were measured using CMC, xylan, and p-nitrophenyl-β-d-cellobioside as substrates, respectively. The reducing sugar released upon the hydrolysis of sugar polymers was quantified by dinitrosalicylic acid (DNSA) reagent at 540 nm. One unit of enzymatic activity was defined as the amount of enzyme that released 1 μmol of reducing sugar from the substrate per minute. From the same supernatant sample, protein concentration was estimated using the bicinchoninic acid assay (BCA) reagent kit (GE Healthcare) with bovine serum albumin as the standard.

### RNA isolation

Cells were harvested at 36 h for glucose and 60 h for all the other substrates, such as Avicel, wheat bran, Avicel + wheat bran, and biomass. The mycelium was filtered using autoclaved Miracloth, washed several times with sterile distilled water and stored at − 80 °C until RNA extraction. Total RNA was extracted using the Qiagen RNeasy Mini Kit for Plants and Fungi with on-column treatment with RNase-Free DNase (Qiagen) as per the manufacturer’s instructions. Sample integrity was confirmed on agarose gel, and quality was measured using the 260/280 nm ratio.

### Transcriptome sequencing and assembly

Paired-end Illumina mRNA libraries were generated from total RNA samples from fungal mycelia cultivated in different carbon substrates in accordance with the manufacturer’s instructions (Illumina Inc., USA). Transcriptome sequencing was performed on the Illumina HiSeq2000 platform (Centre for Cellular and Molecular Platforms, Bangalore, India). The raw reads were pre-processed by removing adaptor sequences, and discarding empty reads and low-quality sequences. The high-quality paired end 101 bp reads of each sample were used for transcriptome assembly using TopHat (version 2.1.0; http://tophat.cbcb.umd.edu/) and Cufflinks (version 2.2.1; http://cufflinks.cbcb.umd.edu/) for reference-based assembly [51]. Assembly was performed via Cufflinks using the TopHat mapping files with default parameters. The final assembly was obtained by merging the individual assemblies with default options using Cuffmerge. Functional annotation of the assembled transcripts was carried out using BLASTP search with an *E *value cutoff of ≤ 1e−5 against the *P. funiculosum* NCIM1228 predicted proteome sequence.

### Expression analysis using RT-qPCR

100 ng of RNA was used as a template in each quantitative real-time PCR (RT-qPCR) reaction. cDNA synthesis control was performed to ensure the absence of DNA contamination. RT-qPCR was carried out using iTaq™ Universal SYBR® Green Supermix (Bio-Rad) and Bio-Rad CFX96 qPCR detection system. Primers for test and control transcripts were designed using a boundary sequence of two exons to avoid any amplification from genomic DNA contamination (Additional file 8: Table S6). RT-qPCR was done in biological triplicates with actin as the endogenous control. Relative expression levels were normalized to actin, and fold changes in RNA level were the ratios of the relative expression level under repressing conditions (glucose) and cellulase-inducing conditions (Avicel).

### Supplementary Information

**Additional file 1.** Protein coding genes identified in P. funiculosum NCIM1228.

**Additional file 2.** CAZymes identified in P. funiculosum NCIM1228.

**Additional file 3.** Transcription factors identified in P. funiculosum NCIM1228.

**Additional file 4.** The list of the carbon substrates in which each identified CAZymes is detected.

**Additional file 5.** Expression values of CAZymes in all the five carbon substrate corressponding to the heat map.

**Additional file 6.** The list of carbon substrates in which each identified TF is detected.

**Additional file 7.** Expression values of TFs in all the five carbon substrate corressponding to the heat map.

**Additional file 8: Table S1.** *P. funiculosum* NCIM1228 TF homologs. **Table S2.** Summary of read data, mapping and reference-based assembly obtained for each growth condition of *P. funiculosum*. **Table S3.** Significant differential expression of TFs. **Table S4.** Expression levels of TFs through RT-qPCR and Illumina RNA-Seq. **Table S5.** Comparative analysis of genome features of filamentous fungi. **Table S6.** Primers for RT-PCR. **Figure S1.** Domain architecture of *P. funiculosum* NCIM1228 CAZy family proteins. **Figure S2.** Expression analysis along with the domain architecture of *P. funiculosum* NCIM1228 TFs. **Figure S3.** Cellulolytic activities and supernatant protein estimation of *P. funiculosum* NCIM1228. **Figure S4.** Correlation of RNA-Seq Data obtained from the biological replicates. **(A)** Graphs representing the Pearson correlation between biological replicates of each sample. A high Pearson correlation was obtained demonstrating the reliability of RNA-seq analysis (R ≥ 0.95). **(B)** Boxplot of all normalized samples showing that all samples and conditions are comparable. **Figure S5.** Principal component analysis (PCA) of gene expression levels in A) CAZymes and B) TFs in replicates of the five carbon substrates—Glucose, Avicel, Wheat bran, Avicel + wheat bran, and biomass. **Figure S6.** Expression analysis of TF coding genes in response to crystalline carbon substrate Avicel using RT-qPCR. **Figure S7.** Alignment of transcription factors with the known homologs.

## Data Availability

This Whole Genome Shotgun project has been deposited at DDBJ/ENA/GenBank under the accession JAVBHS000000000. The version described in this paper is version JAVBHS010000000.
