# Supplementary material for: Systematic identification of CAZymes and transcription factors in the hypercellulolytic fungus Penicillium funiculosum NCIM1228 involved in lignocellulosic biomass degradation
Source: Biotechnol Biofuels Bioprod. 2023 Oct 4;16:150. doi: 10.1186/s13068-023-02399-9 (PMC10552389; doi:10.1186/s13068-023-02399-9)
Supplement: Supplementary file 8 — Additional file 8: Table S1. P. funiculosum NCIM1228 TF homologs. Table S2. Summary of read data, mapping and reference-based assembly obtained for each growth condition of P. funiculosum. Table S3. Significant differential expression of TFs. Table S4. Expression levels of TFs through RT-qPCR and Illumina RNA-Seq. Table S5. Comparative analysis of genome features of filamentous fungi. Table S6. Primers for RT-PCR. Figure S1. Domain architecture of P. funiculosum NCIM1228 CAZy family proteins. Figure S2. Expression analysis along with the domain architecture of P. funiculosum NCIM1228 TFs. Figure S3. Cellulolytic activities and supernatant protein estimation of P. funiculosum NCIM1228. Figure S4. Correlation of RNA-Seq Data obtained from the biological replicates. (A) Graphs representing the Pearson correlation between biological replicates of each sample. A high Pearson correlation was obtained demonstrating the reliability of RNA-seq analysis (R ≥ 0.95). (B) Boxplot of all normalized samples showing that all samples and conditions are comparable. Figure S5. Principal component analysis (PCA) of gene expression levels in A) CAZymes and B) TFs in replicates of the five carbon substrates—Glucose, Avicel, Wheat bran, Avicel + wheat bran, and biomass. Figure S6. Expression analysis of TF coding genes in response to crystalline carbon substrate Avicel using RT-qPCR. Figure S7. Alignment of transcription factors with the known homologs. [file 13068_2023_2399_MOESM8_ESM.docx]

| **qseqid** | **sseqid** | **pident** | **length** | **mismatch** | **qstart** | **qend** | **sstart** | **send** | **evalue** | **bitscore** |
| --- | --- | --- | --- | --- | --- | --- | --- | --- | --- | --- |
| AreA | C0067G0.23 | 53.027 | 892 | 291 | 59 | 876 | 6 | 843 | 0 | 735 |
| AraR | C0006G0.30 | 71.144 | 603 | 157 | 236 | 832 | 1 | 592 | 0 | 814 |
| AmyR | C0012G2.24 | 48.718 | 390 | 175 | 141 | 522 | 9 | 381 | 1.09E-111 | 339 |
| BrlA | C0016G0.20 | 58.042 | 429 | 163 | 7 | 431 | 6 | 421 | 4.29E-159 | 454 |
| ClrB | C0021G3.75 | 46.834 | 758 | 342 | 1 | 730 | 1 | 725 | 0 | 615 |
| ClrC | C0127G0.8 | 34.862 | 327 | 146 | 4 | 296 | 33 | 326 | 2.65E-35 | 129 |
| Hap2 | C0020G2.1 | 64.286 | 364 | 119 | 1 | 360 | 1 | 357 | 1.39E-128 | 371 |
| Hap3 | C0029G1.88 | 70.093 | 214 | 53 | 4 | 207 | 2 | 214 | 5.07E-97 | 280 |
| Hap5 | C0099G0.143 | 80.672 | 238 | 36 | 16 | 249 | 29 | 260 | 1.81E-126 | 359 |
| PacC | C0040G1.97 | 38.501 | 587 | 275 | 93 | 620 | 104 | 663 | 5.00E-85 | 278 |
| FlbC | C0019G3.93 | 65.084 | 358 | 101 | 1 | 347 | 1 | 345 | 2.14E-139 | 398 |
| Rca1 | C0037G1.47 | 43.407 | 182 | 80 | 5 | 165 | 3 | 182 | 1.11E-39 | 140 |
| Vib1 | C0088G0.11 | 46.4 | 250 | 115 | 129 | 363 | 89 | 334 | 3.36E-66 | 227 |
| XlnR | C0081G0.41 | 63.207 | 973 | 270 | 28 | 987 | 1 | 898 | 0 | 1093 |

**Table S1. *P. funiculosum* NCIM1228 TF homologs**

**Table S2. Summary of read data, mapping and reference-based assembly obtained for each growth condition of *P. funiculosum***

| **Sample name** | **Replicate** | **Total reads** | **High quality reads** | **Mapped reads** |
| --- | --- | --- | --- | --- |
| **Glucose** | **R1** | 85784648 | 76550298 | 75018290  (98%) |
|  | **R2** | 67714212 | 64769376 | 62408749 (96.36%) |
| **Avicel & Wheat Bran** | **R1** | 59101874 | 53004944 | 50781204 (95.8%) |
|  | **R2** | 49936412 | 47622140 | 45731102 (96.03%) |
| **Avicel** | **R1** | 92573276 | 82075640 | 80491002 (98.06%) |
|  | **R2** | 65933432 | 62467836 | 60127053 (96.25%) |
| **Wheat Bran** | **R1** | 67286514 | 60093304 | 58240998 (96.91) |
|  | **R2** | 48018034 | 47622140 | 43770991 (96.22%) |
| **Wheat Straw** | **R1** | 53014004 | 47919816 | 47033864 (98.15%) |
|  | **R2** | 75180772 | 70972282 | 68116423 (95.98%) |

**Table S3. Significant differential expression of TFs**

| **S. no.** | **Exclusive in Glucose-Biomass:** | | | | | |  |  |  |  |
| --- | --- | --- | --- | --- | --- | --- | --- | --- | --- | --- |
|  | TF ID | TF domain | Glucose | | Biomass | |  |  |  |  |
| 1 | C0059G0.38 | Zn_clus | 3.85 | 5.51 | 40.06 | 39.14 |  |  |  |  |
| 2 | C0067G0.11 | Fungal_trans_2 | 0 | 0 | 1.056 | 0.641 |  |  |  |  |
| 3 | C0001G9.9 | zf-U1 | 0 | 0 | 22.77 | 1.014 |  |  |  |  |
|  | **Exclusive in Glucose-Avicel:** | | | | | |  |  |  |  |
|  | TF ID | TF domain | Glucose | | Avicel | |  |  |  |  |
| 1 | C0092G0.56 | Fungal_trans_2 | 12.09 | 13.94 | 58.77 | 61.43 |  |  |  |  |
|  | **Exclusive in Glucose-WB:** | | | | | |  |  |  |  |
|  | TF ID | TF domain | Glucose | | WB | |  |  |  |  |
| 1 | C0011G2.116 | Zn_clus | 1.38 | 0.389 | 0.0001 | 0.83 |  |  |  |  |
| 2 | C0001G7.7 | Fungal_trans | 13.21 | 14.36 | 0 | 0 |  |  |  |  |
| 3 | C0040G1.97 | zf-H2C2_2 | 74.19 | 70.30 | 16.46 | 16.70 |  |  |  |  |
|  | **Exclusive in Glucose-WB+Avi:** | | | | | |  |  |  |  |
|  | TF ID | TF domain | Glucose | | WB+Avi | |  |  |  |  |
| 1 | C0021G2.92 | Zn_clus | 0 | 0 | 7.11 | 4.44 |  |  |  |  |
| 2 | C0123G0.32 | Zn_clus | 0 | 0 | 2.31 | 2.87 |  |  |  |  |
| 3 | C0009G4.243 | MFS_1 | 1.12 | 1.16 | 5.50 | 5.06 |  |  |  |  |
| 4 | C0006G2.0 | Fungal_trans | 1.88 | 2.66 | 14.40 | 16.31 |  |  |  |  |
| 5 | C0077G0.36 | Fungal_trans | 1.48 | 1.32 | 0 | 0 |  |  |  |  |
| 6 | C0118G0.63 | Fungal_trans_2 | 2.19 | 2.90 | 18.40 | 18.83 |  |  |  |  |
| 7 | C0029G0.82 | Fungal_trans | 1.26 | 1.36 | 15.83 | 16.15 |  |  |  |  |
| 8 | C0029G2.41 | Myb_DNA-binding | 3.07 | 3.22 | 26.61 | 25.85 |  |  |  |  |
| 9 | C0031G2.62 | HTH_Tnp_Tc5 | 9.32 | 8.43 | 46.68 | 44.66 |  |  |  |  |
|  | **Common in Glucose-WB+Avi and Glucose-Biomass:** | | | | | | | |  |  |
|  | TF ID | TF domain | Glucose | | WB+Avi | | Biomass | |  |  |
| 1 | C0102G0.10 | Zn_clus | 0 | 0 | 0.46 | 0.51 | 0.968 | 0.54 |  |  |
| 2 | C0060G1.70 | Fungal_trans | 98.71 | 142.68 | 21.89 | 24.78 | 22.78 | 25.54 |  |  |
|  | **Common in Glucose-Avicel and Glucose-WB+Avi:** | | | | | | | |  |  |
|  | TF ID | TF domain | Glucose | | Avi | | WB+Avi | |  |  |
| 1 | C0006G5.22 | Fungal_trans_2 | 3.25 | 6.17 | 76.55 | 117.37 | 35.99 | 35.24 |  |  |
|  | **Common in Glucose-Avicel, Glucose-Biomass, and Glucose-WB+Avi:** | | | | | | | | | |
|  | TF ID | TF domain | Glucose | | Avicel | | Biomass | | WB+Avi | |
| 1 | C0016G2.76 | bZIP_1 | 18.31 | 22.05 | 320.06 | 258.09 | 191.67 | 176.69 | 146.72 | 127.06 |
| 2 | C0005G2.0 | Fungal_trans | 104.96 | 190.14 | 25.83 | 30.8 | 23.39 | 24.14 | 20.63 | 22.09 |
| 3 | C0097G0.92 | Fungal_trans | 0 | 0 | 2.478 | 2.17 | 8.87 | 5.07 | 3.91 | 5.18 |

**Table S4: Expression levels of TFs through RT-qPCR and Illumina RNA-Seq**

| **S.no.** | **TF** | **log2 fold change in Avicel wrt glucose** | | |
| --- | --- | --- | --- | --- |
|  |  | **RT-qPCR** | **RNA Sequencing Run1** | **RNA Sequencing Run2** |
| 1 | C0006G5.22 | 1.45 | 4.25 | 4.56 |
| 2 | C0108G0.26 | 4.20 | 3.31 | 2.93 |
| 3 | C0016G2.76 | 3.20 | 3.55 | 4.13 |
| 4 | C0117G0.9 | 2.01 | 0.81 | 0.92 |
| 5 | C0065G0.132 | -2.75 | -0.90 | -1.44 |
| 6 | C0115G0.74 | 1.67 | 1.34 | 1.88 |
| 7 | C0092G0.56 | 1.85 | 2.14 | 2.28 |
| 8 | C0106G0.10 | 0.71 | 0.41 | 0.58 |
| 9 | C0020G3.17 | 1.08 | 4.62 | 3.03 |
| 10 | C0059G0.38 | 3.68 | 1.49 | 1.90 |
| 11 | C0109G0.28 | 2.84 | 1.06 | 1.55 |
| 12 | C0063G0.151 | 1.98 | 2.46 | 3.33 |
| 13 | C0019G3.7 | 1.12 | 3.24 | 3.40 |
| 14 | C0011G3.21 | 1.68 | 1.58 | 1.59 |

**Table S5. Comparative analysis of genome features of filamentous fungi**

| **S. no.** | **Fungus** | **Genome size (Mb)** | **Protein coding genes** | **Reference** |
| --- | --- | --- | --- | --- |
| 1 | *Penicillium funciculosum* NCIM 1228 | 37.75 | 10,739 | This manuscript |
| 2 | *Talaromyces funiculosus* X33 | 28.49 | Not available | NCBI |
| 3 | *Talaromyces pinophilus* | 36.51 | 13,472 | 39 |
| 4 | *Penicillium* *chrysogenum* 54-1255 | 32.19 | 12,943 | 52 |
| 5 | *Talaromyces cellulolyticus* | 36.4 | 10,980 | 53 |
| 6 | *Myceliophthora thermophila* | 38.7 | 9,110 | 54 |
| 7 | *Trichoderma reesei* QM6a | 34.92 | 10,877 | 55 |
| 8 | *Trichoderma reesei* rutc30 | 32.7 | 9,852 | 56 |
| 9 | *Penicillium janthinellum* NCIM1366 | 37.6 | 11,848 | 57 |
| 10 | *Penicillium subrubescens* | 39.75 | 14,188 | 58 |
| 11 | *Penicillium purpurogenum* | 36.2 | 11,057 | 59 |
| 12 | *Penicillium decumbens* | 30.19 | 10,021 | 60 |

**Table S6. Primers for RT-PCR**

| **S. no.** | **Gene ID** | **Forward Primer Sequence (5'-----3')** | **Reverse Primer Sequence (5'-----3')** |
| --- | --- | --- | --- |
| 1 | C0006G5.22 | TGATCCTGTTCCACTGTTCTG | TTTACCGATCATTGTAGCGCC |
| 2 | C0108G0.26 | AGTGTGCAAGGCTTGTGATC | GTAGAGTTCTTGTAGACCGTT |
| 3 | C0016G2.76 | ATTTCTCGCTTGCTCAACCAG | TTGATGTGCGACTCCAGGTTT |
| 4 | C0117G0.9 | AGCGACCGAACTCTGATCCA | TACAGCGTTGGCCATGCATG |
| 5 | C0065G0.132 | AGTGAGAAGCGATCATGGGC | TATCATTACTTCGAGAGCTAGT |
| 6 | C0115G0.74 | ATGATACGAATGCAGACAATGC | TAGAACTTAGGTGTCACGTAG |
| 7 | C0092G0.56 | ACATGTCCAGTGTACAGAAGA | TAGTATTGATCCAGCGGAACT |
| 8 | C0106G0.10 | AGTCTCAAAACGCAAATGCATC | TCAACATCATCAGGCCTGAG |
| 9 | C0020G3.17 | ACCGACTTGTCTGGCTATCG | TGGTCAGAGGGTAAACGAGA |
| 10 | C0059G0.38 | AGTGCAGCTTCCAGCCTCA | TTGCGTACCATCTGTATCGC |
| 11 | C0109G0.28 | ATGGAGCAACAGAATCAGTTC | GAATGGGATCTTGTCTATGGA |
| 12 | C0063G0.151 | ACACACTCCTCCATCATCTTC | TCAGAGACATGGTCTTGCTTC |
| 13 | C0019G3.7 | TCGTATCATGATGACAATGTGC | TGCCAGAGGTTGCCAGATCT |
| 14 | C0011G3.21 | ATGGACTGCTGCACCACTAT | ATCCGATAGTCTATCAGAGAC |

**A. Domains associated with GH family members**


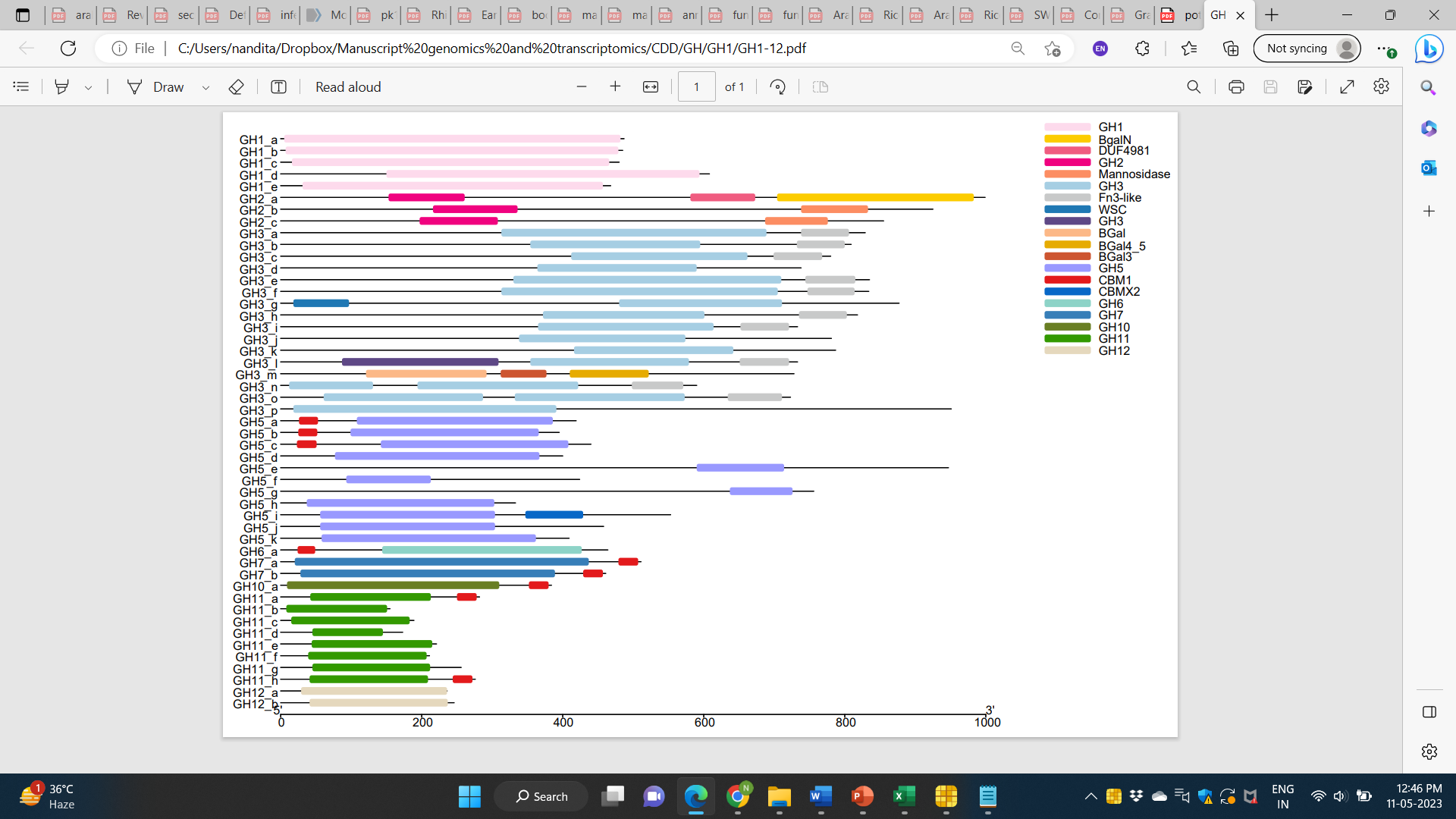

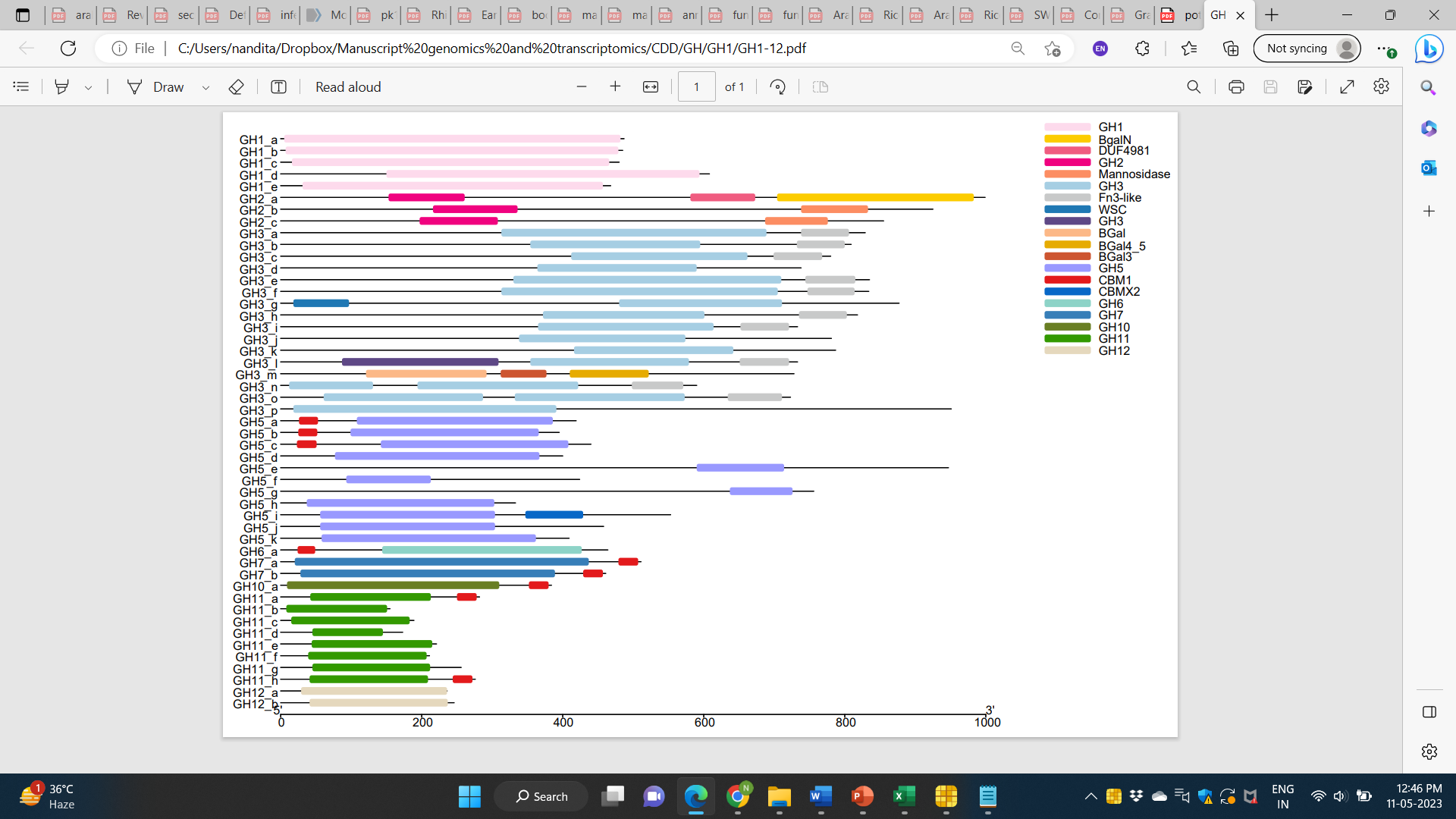

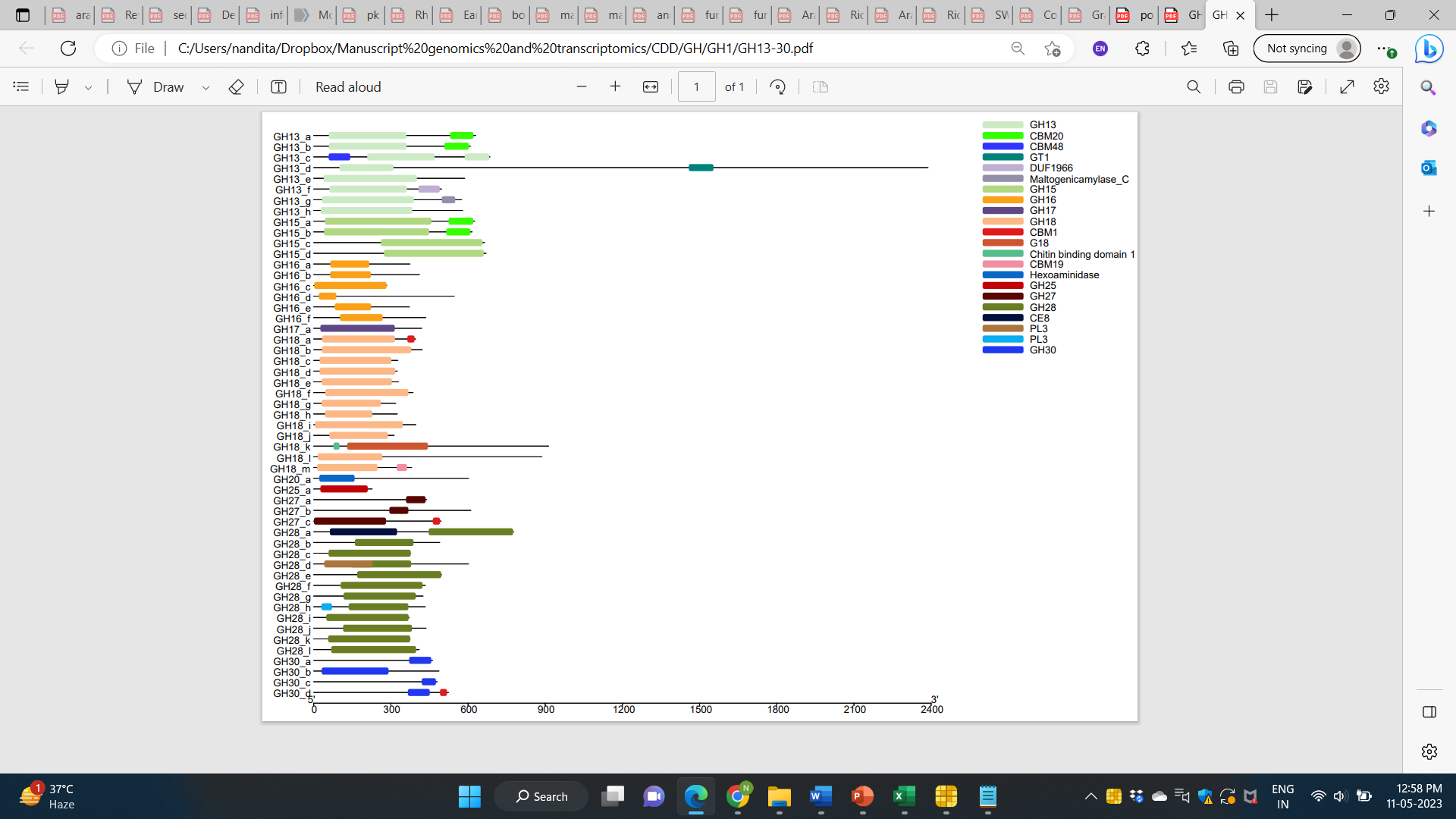

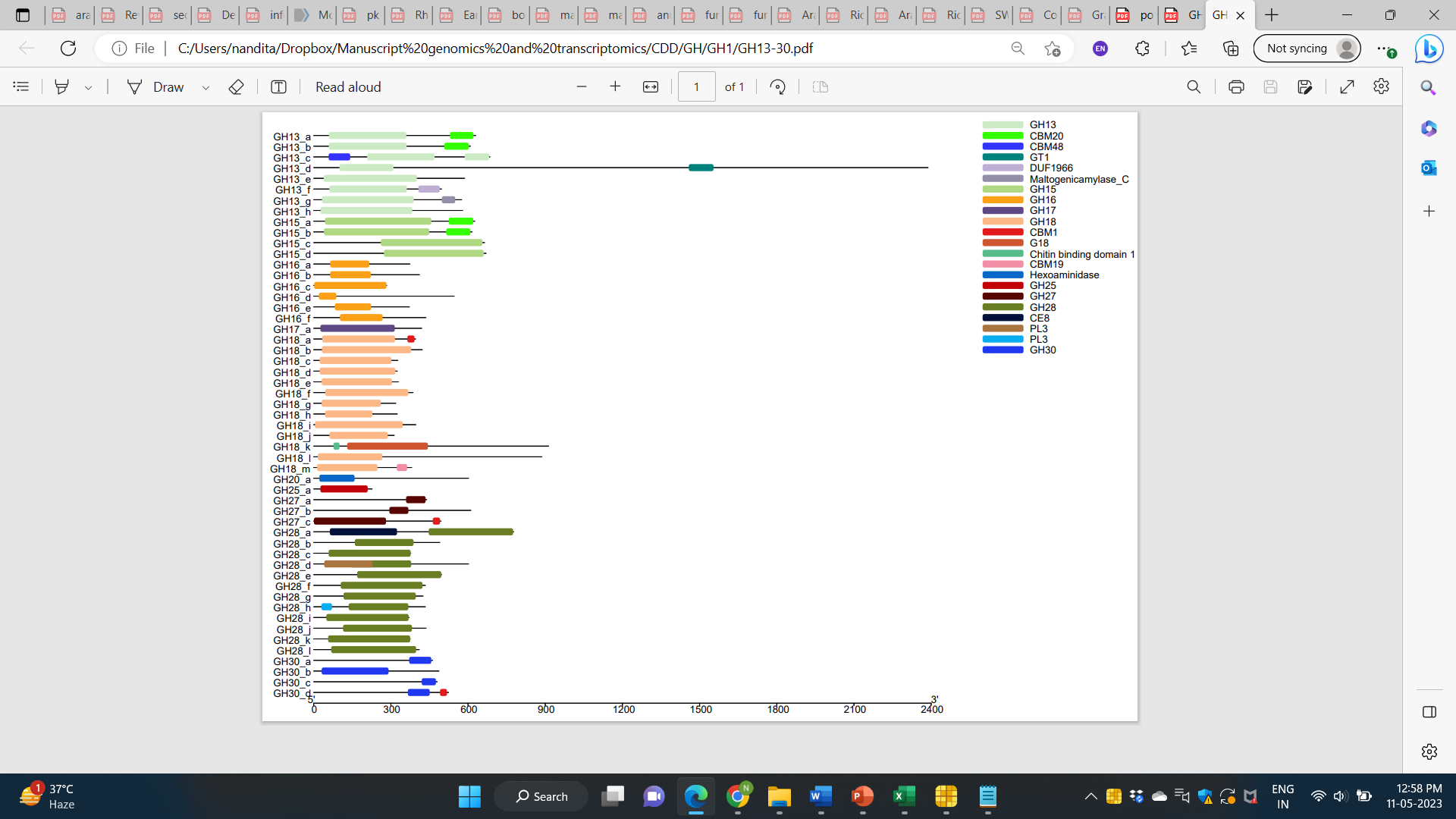

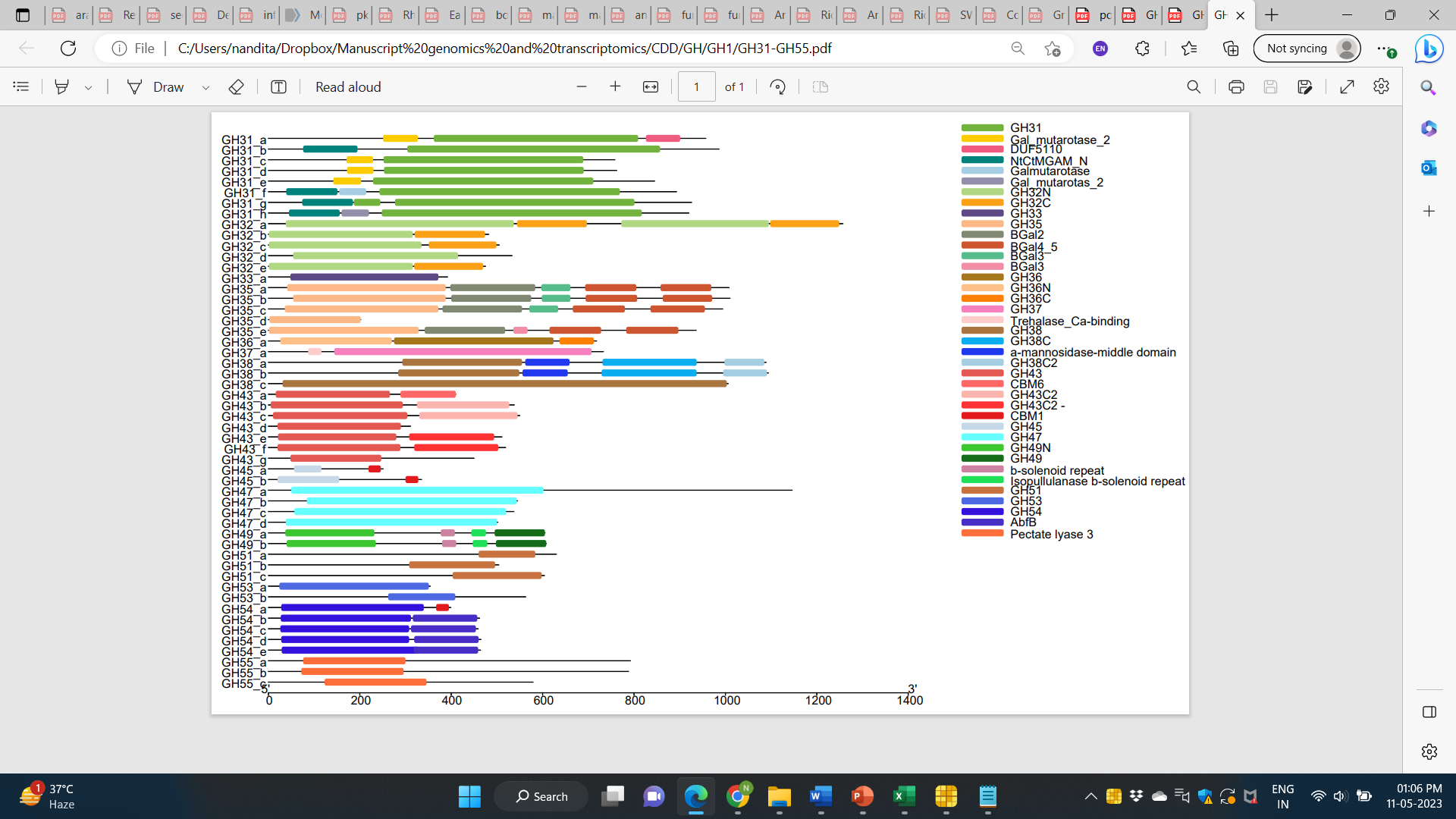

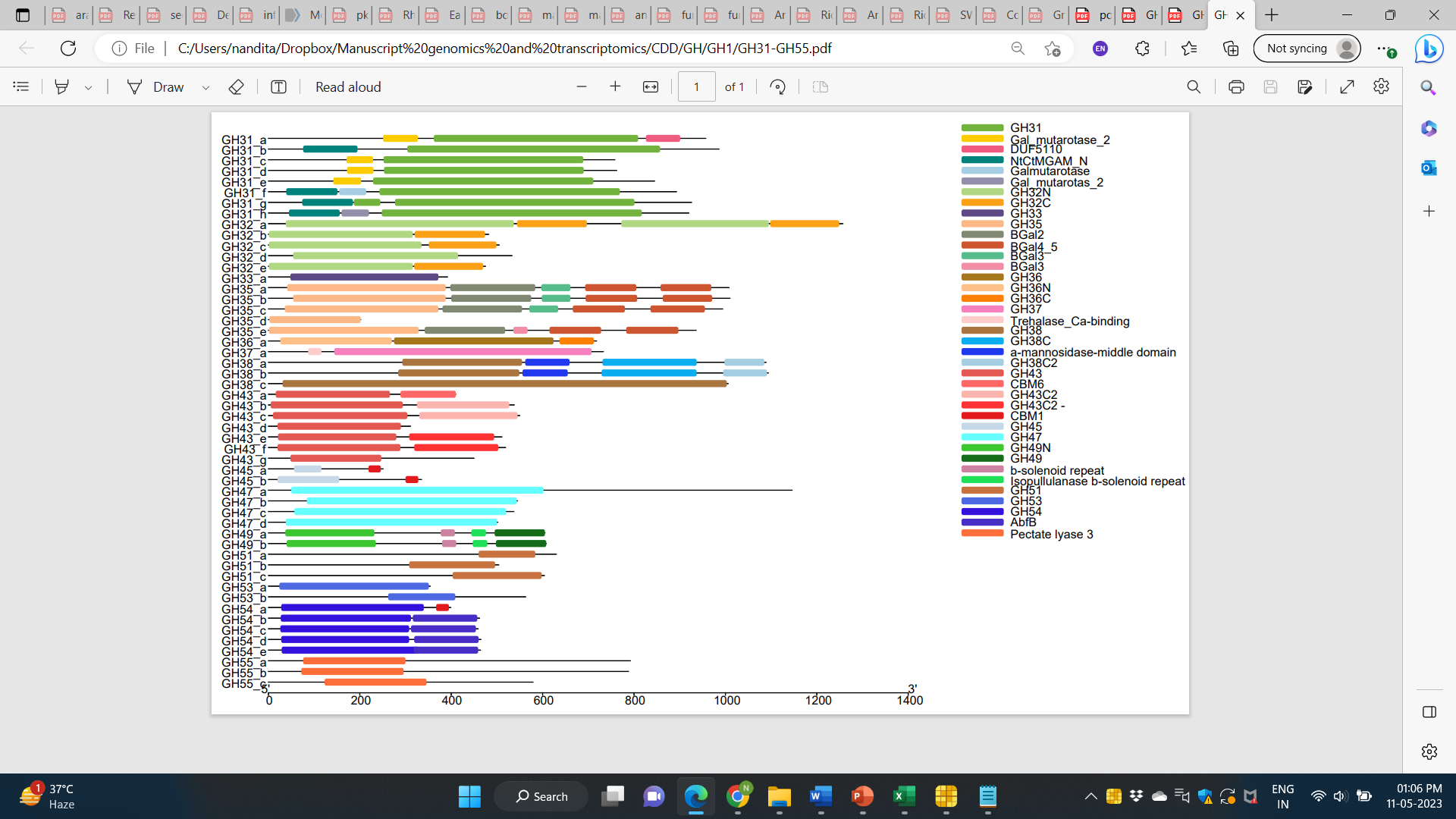

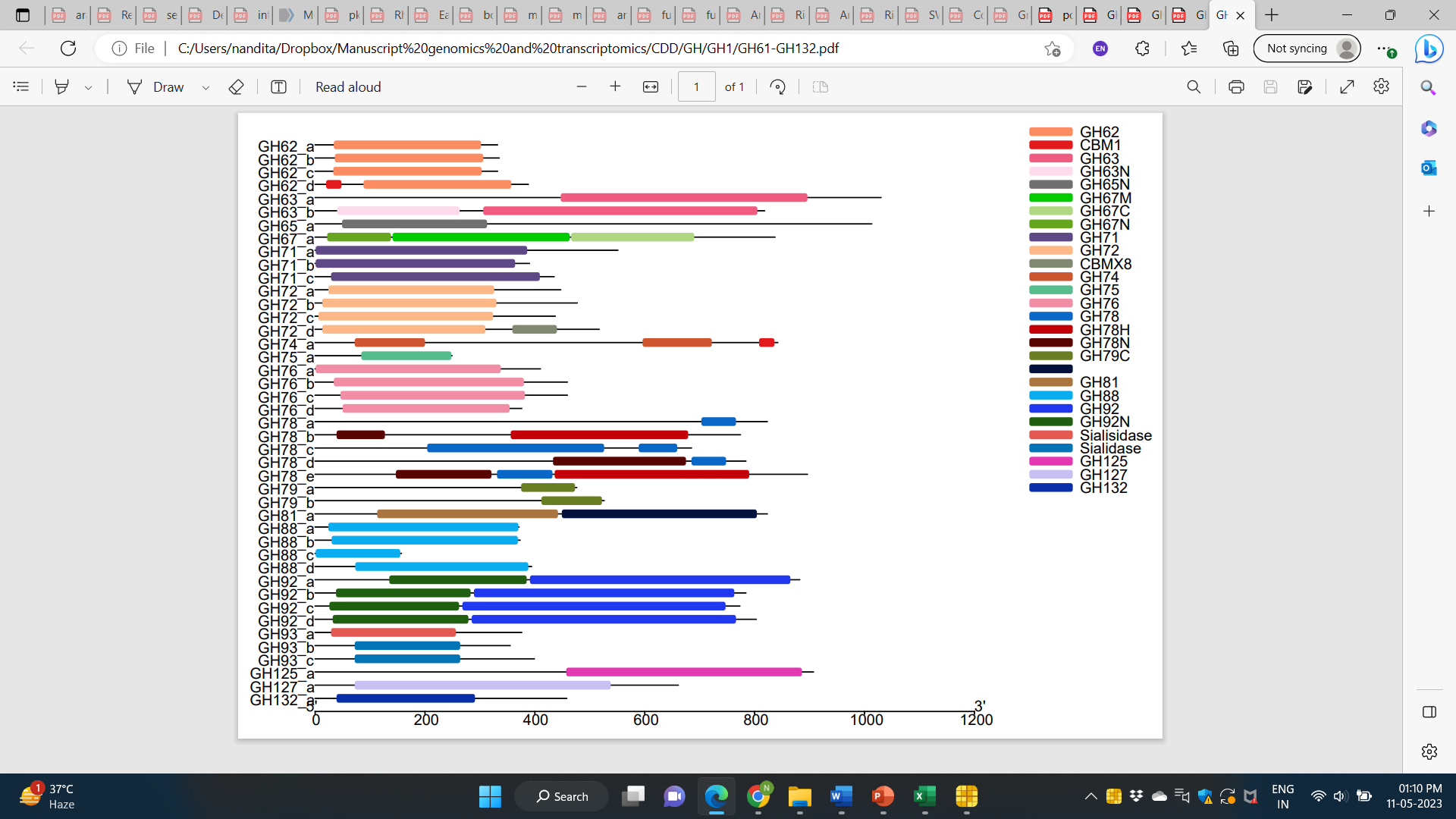

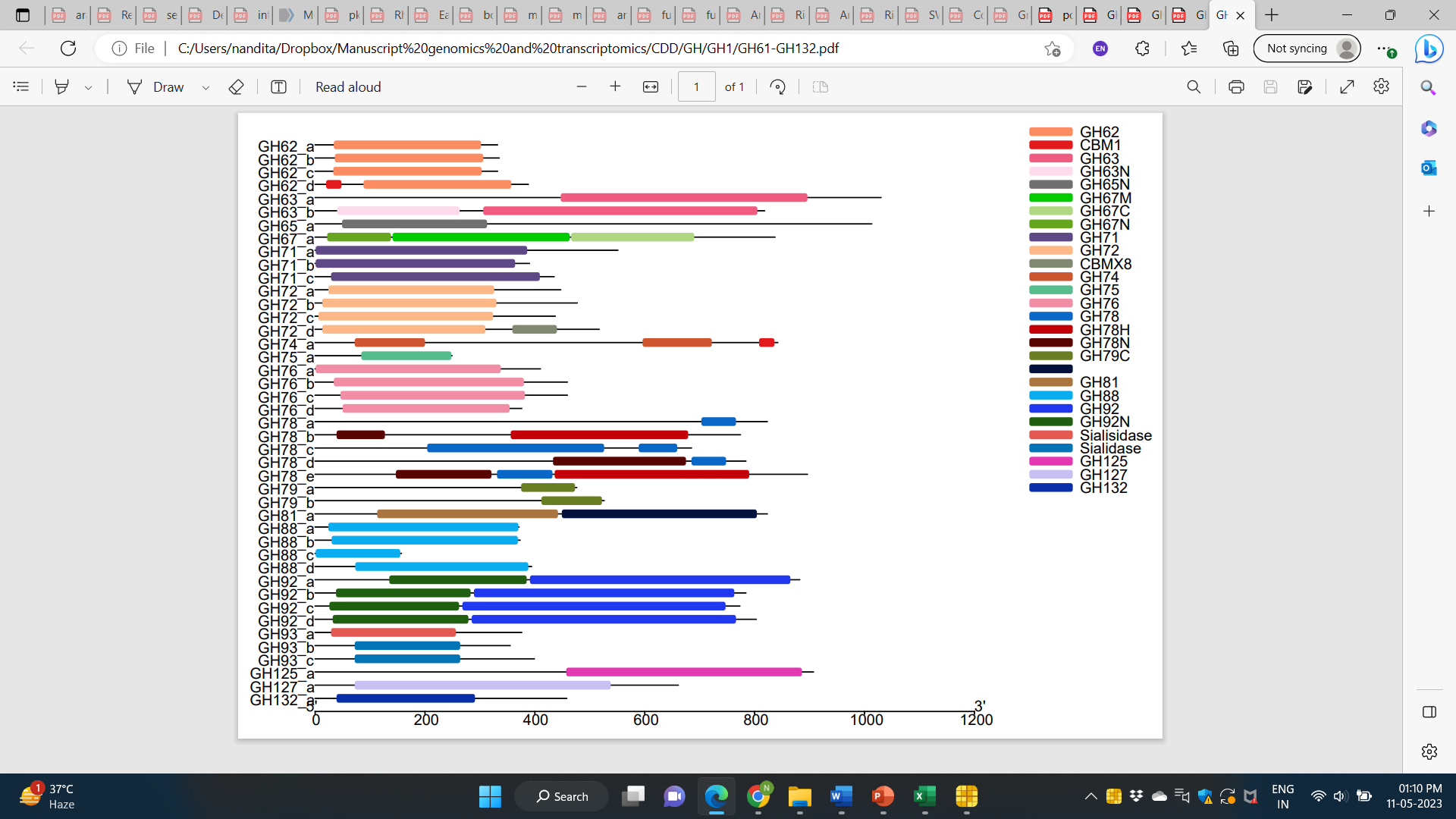


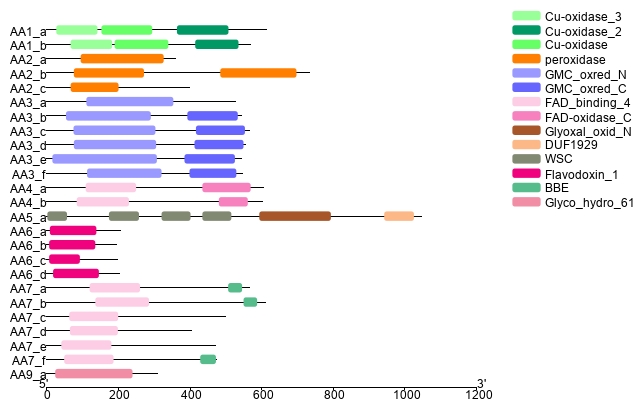


**C. Domains associated with CE family members**

**B. Domains associated with AA family members**


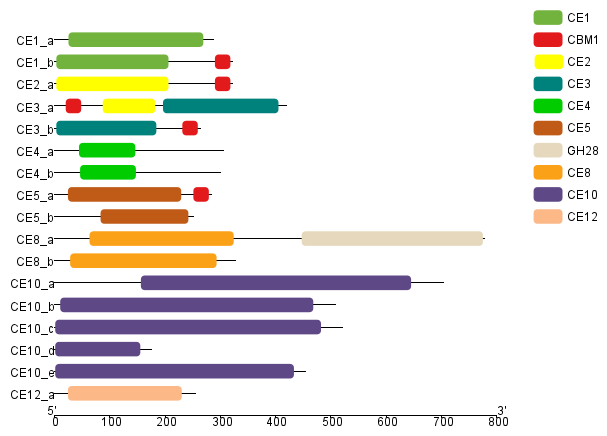


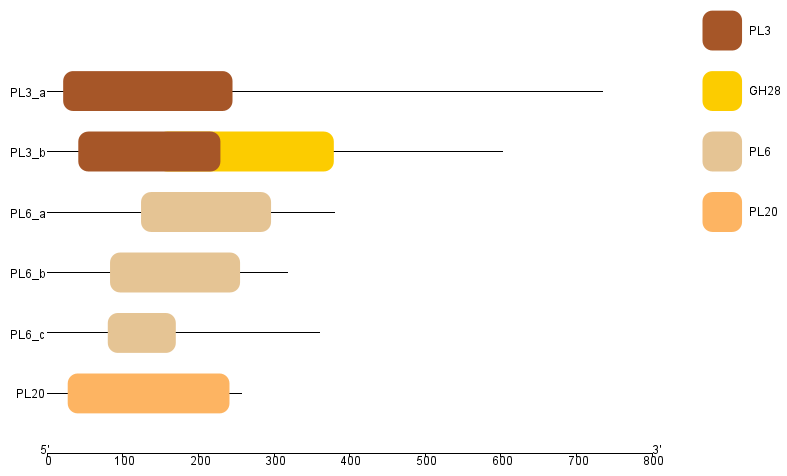


**D. Domains associated with PL family members**


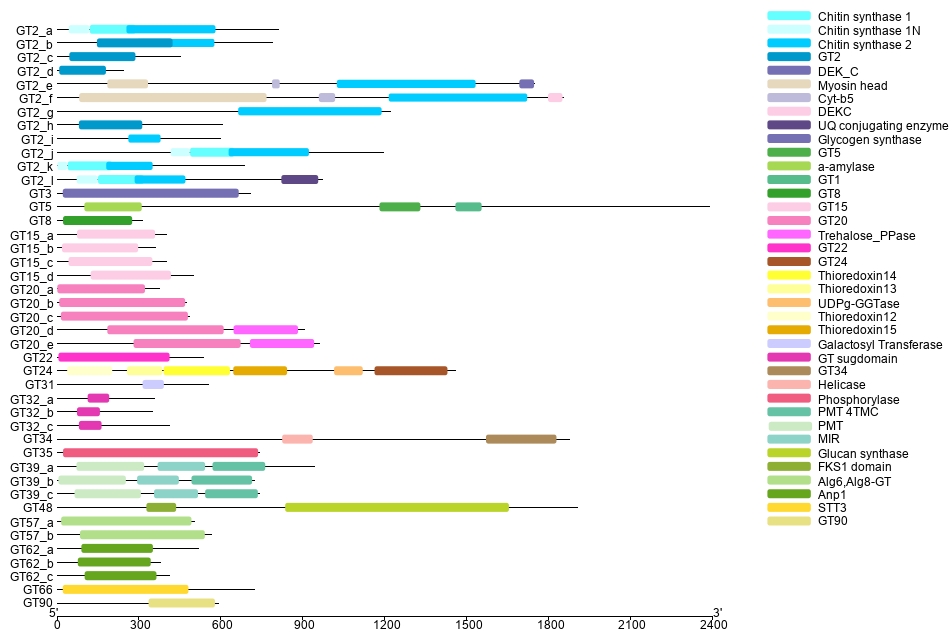

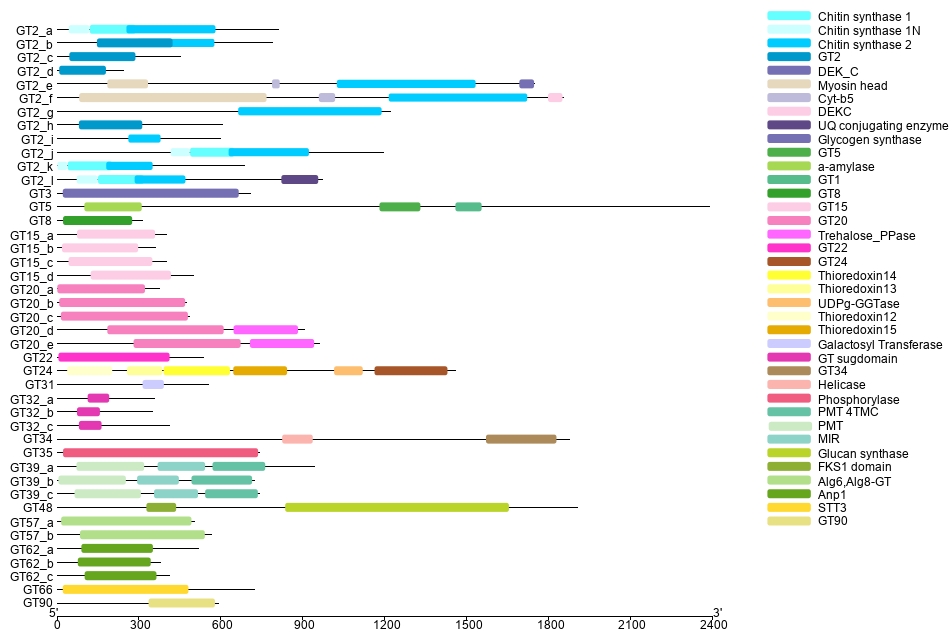


**E. Domains associated with GT family members**

**Figure S1: Domain architecture of *P. funiculosum* NCIM1228 CAZy family proteins**

**A. bZIP_1 family**


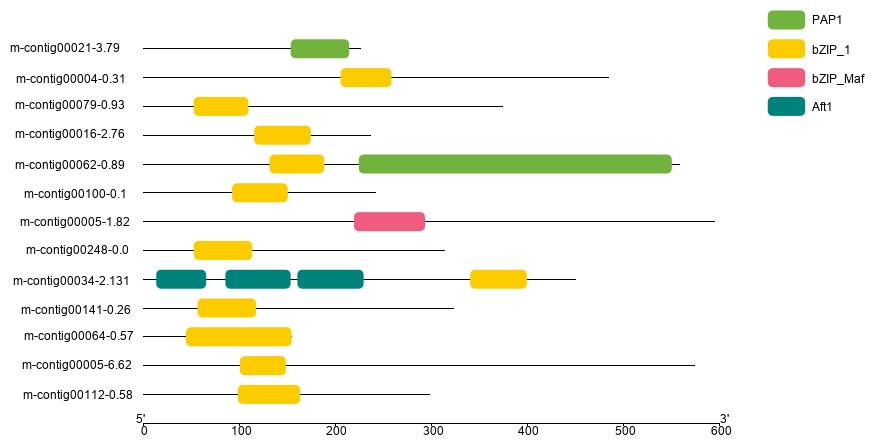

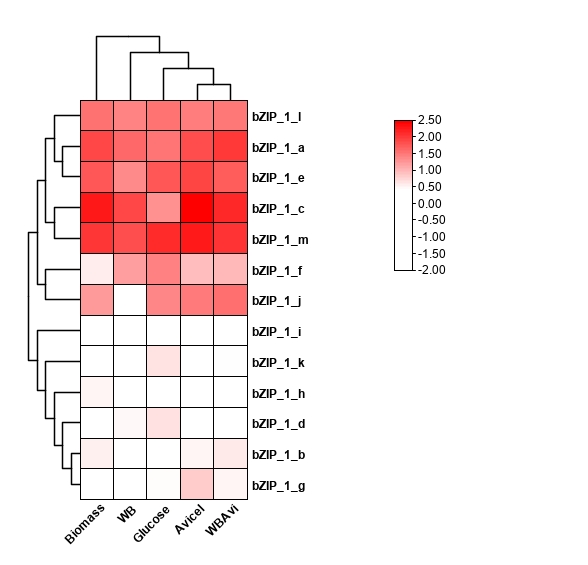

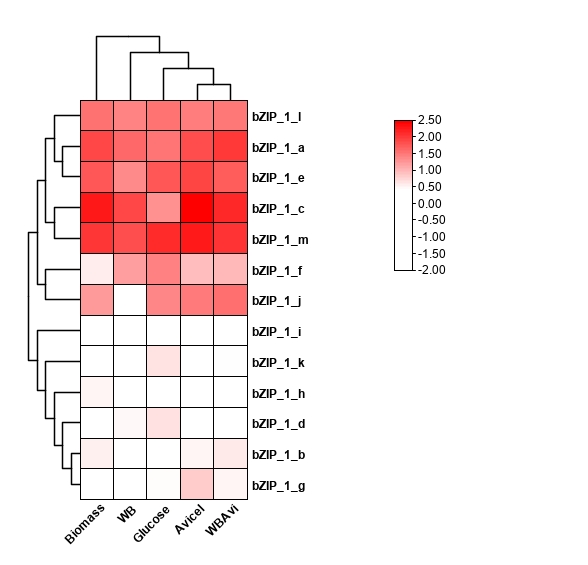

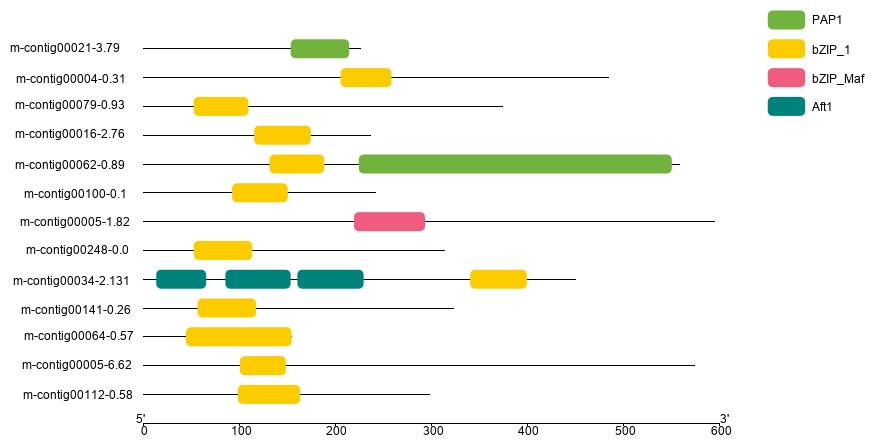


**B. Zn_clus family**


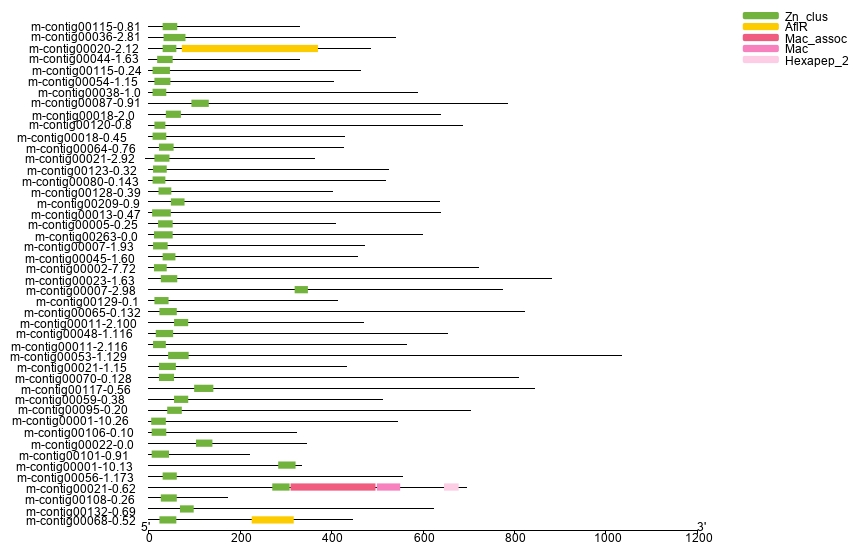

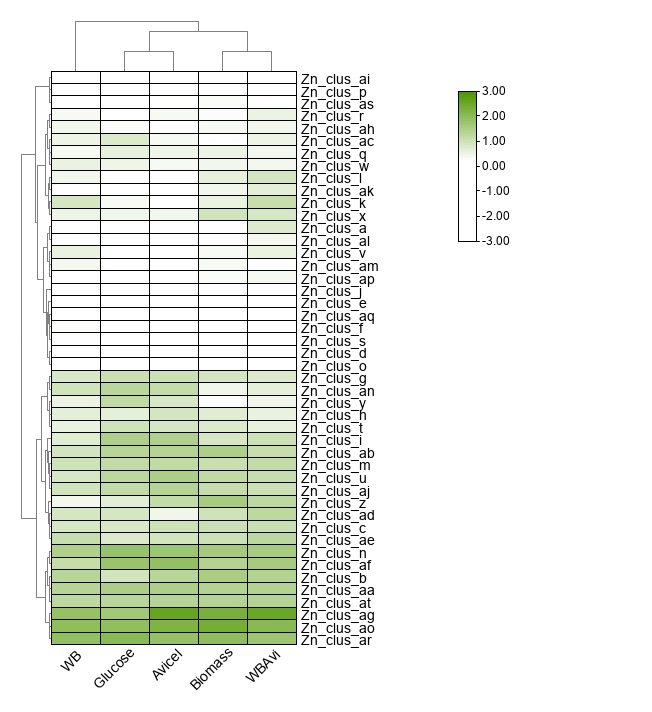

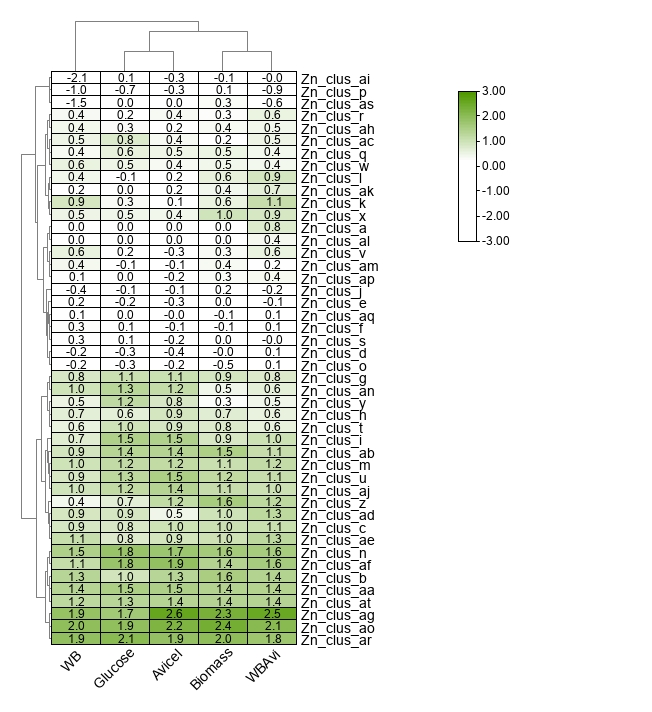

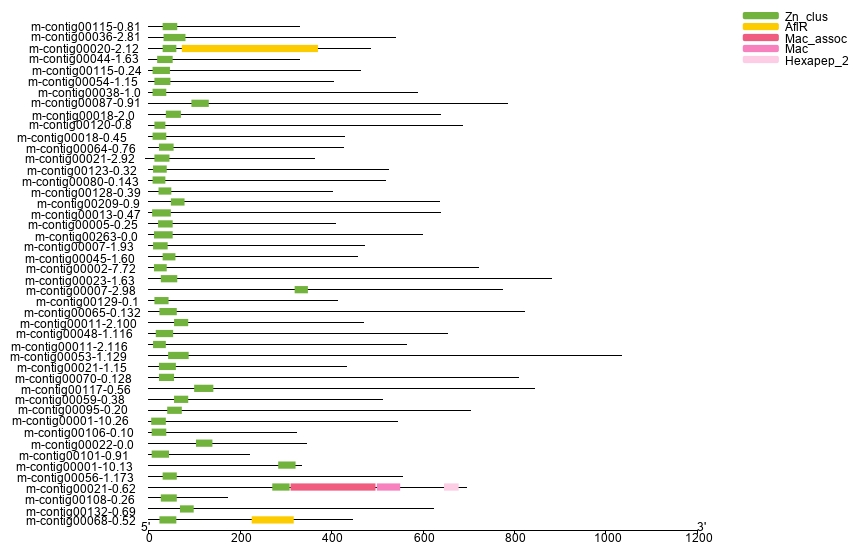


**C. Fungal_trans family**


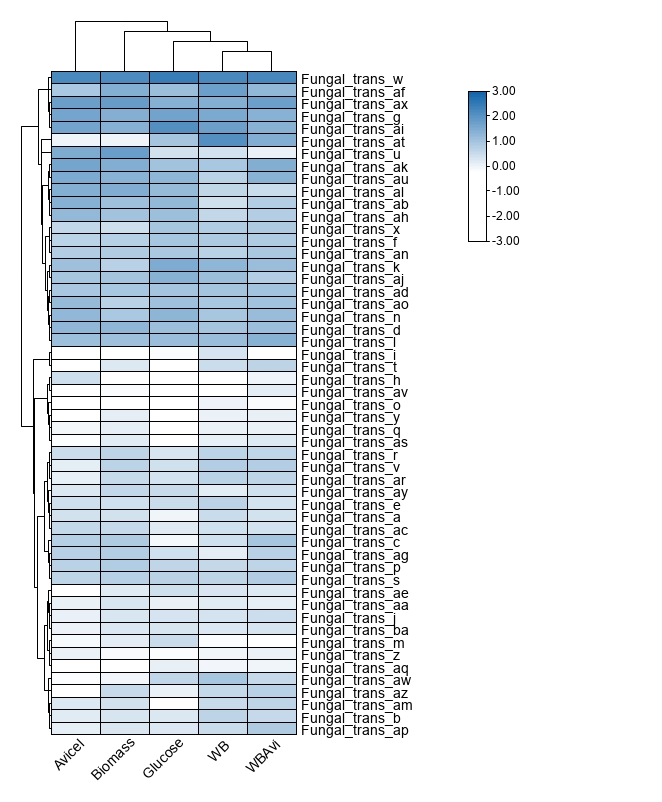


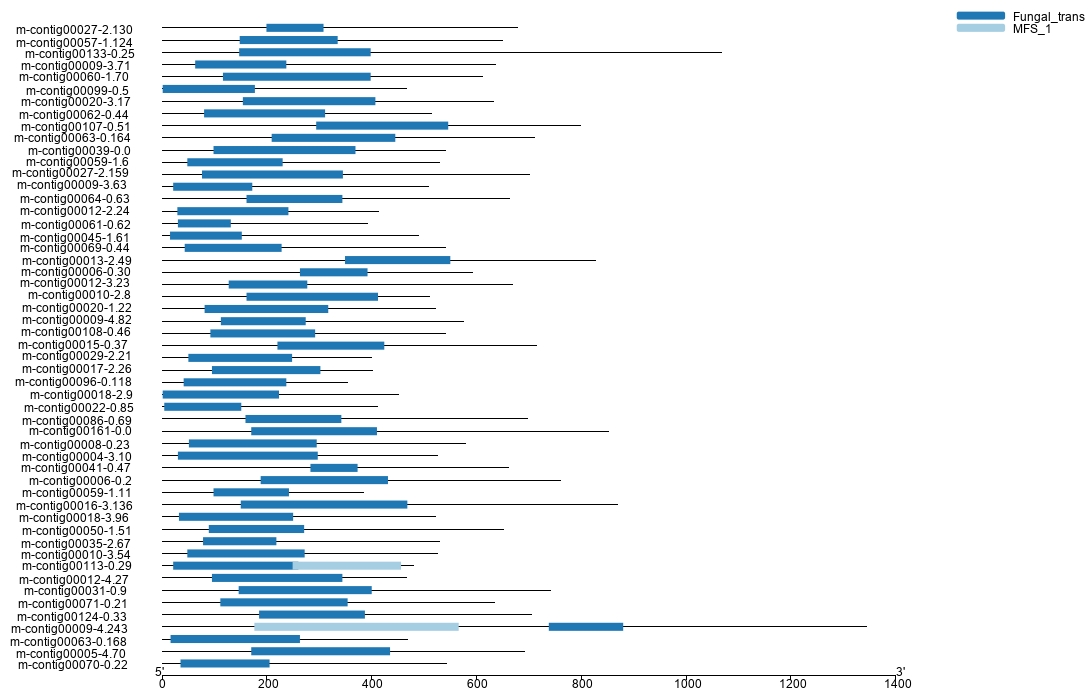

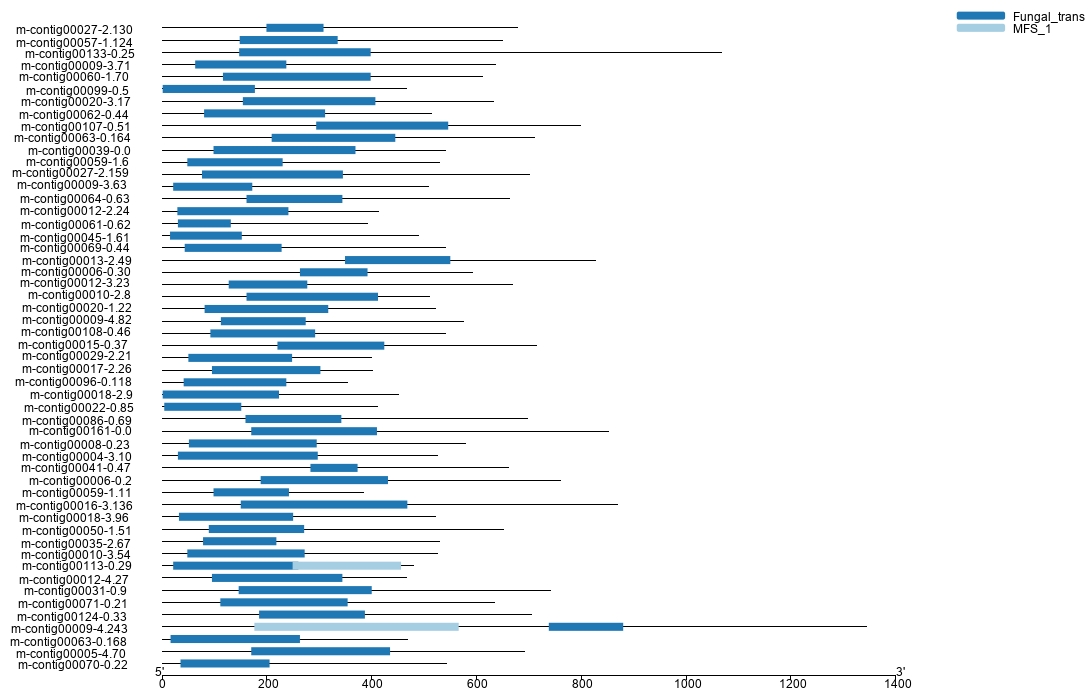

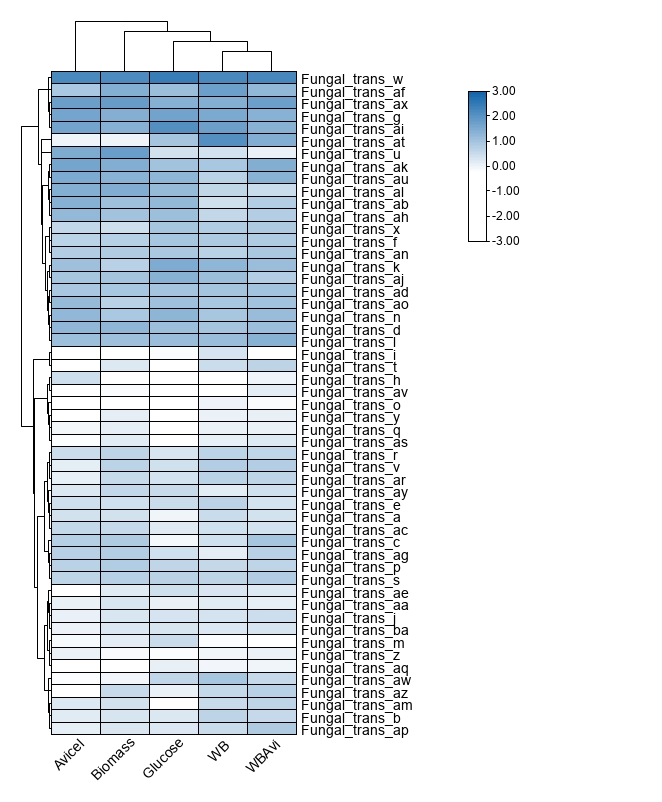


*Fungal_trans_u – relatively low expression in glucose and high in rest

**D. Fungal_trans and ZnF Family**


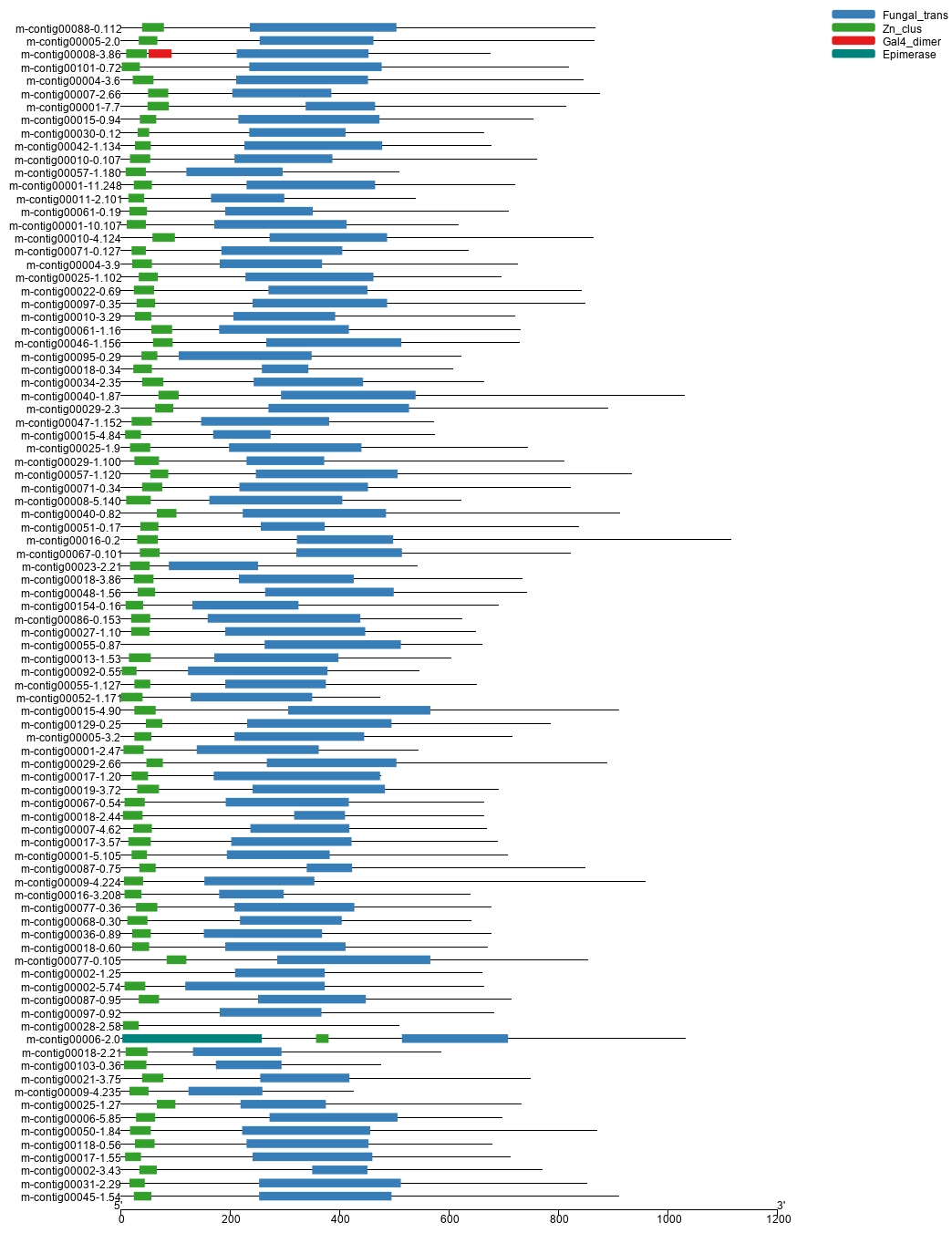

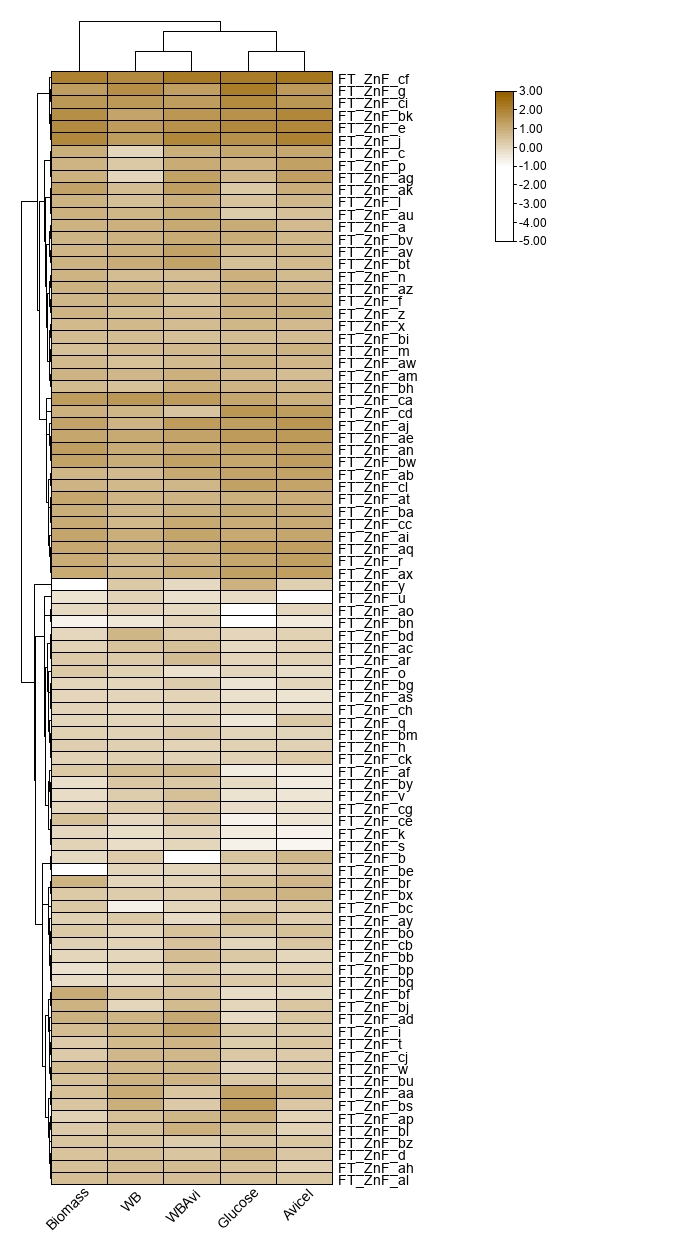

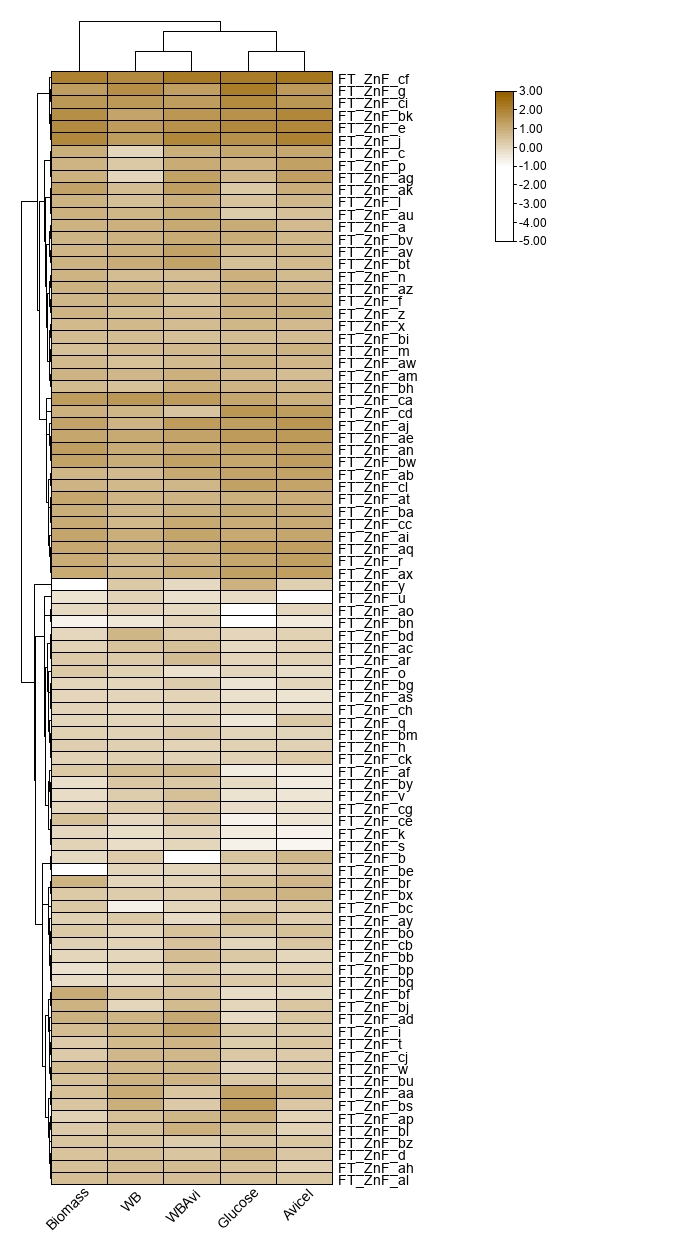

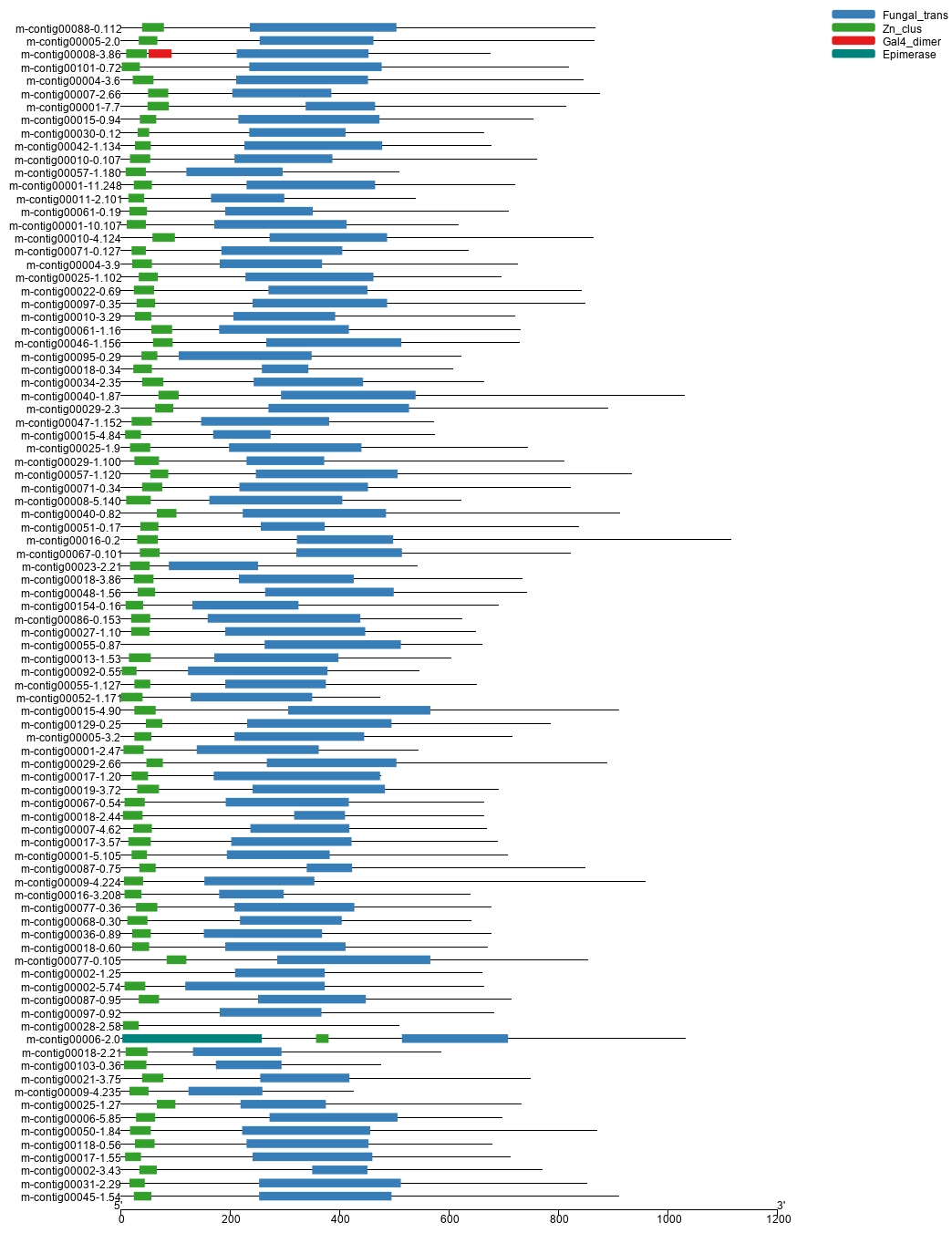


**E. Fungal_trans2_Zn finger family**


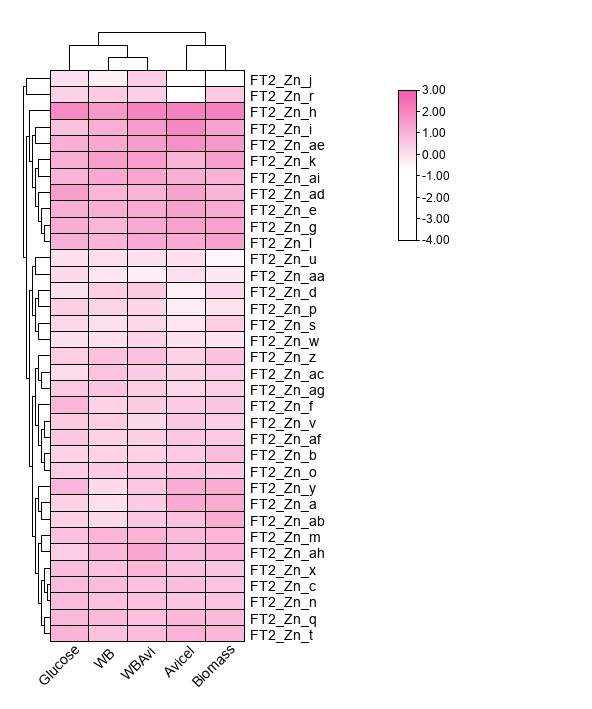


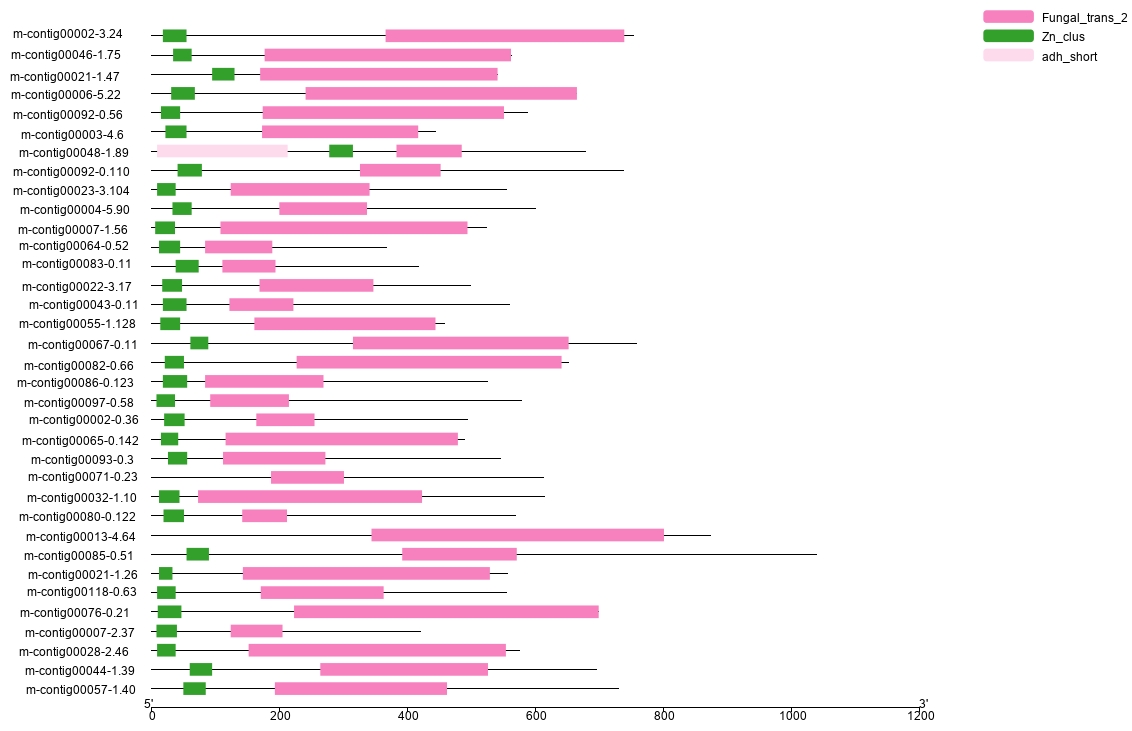


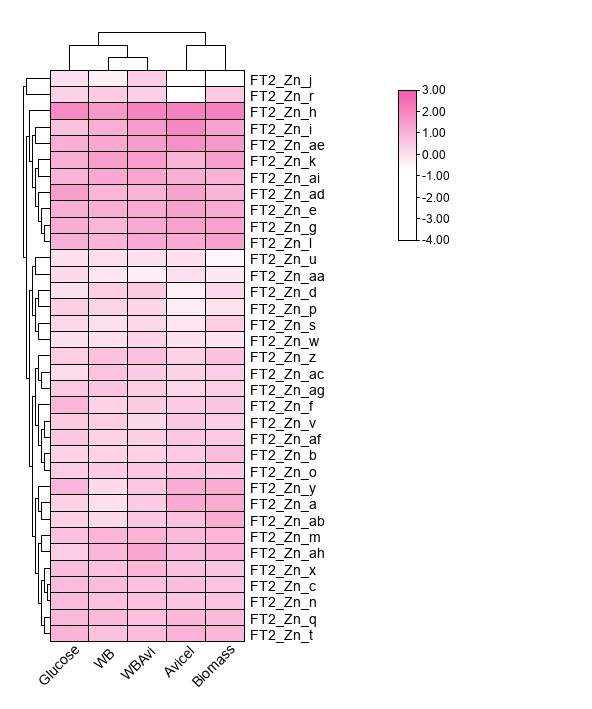


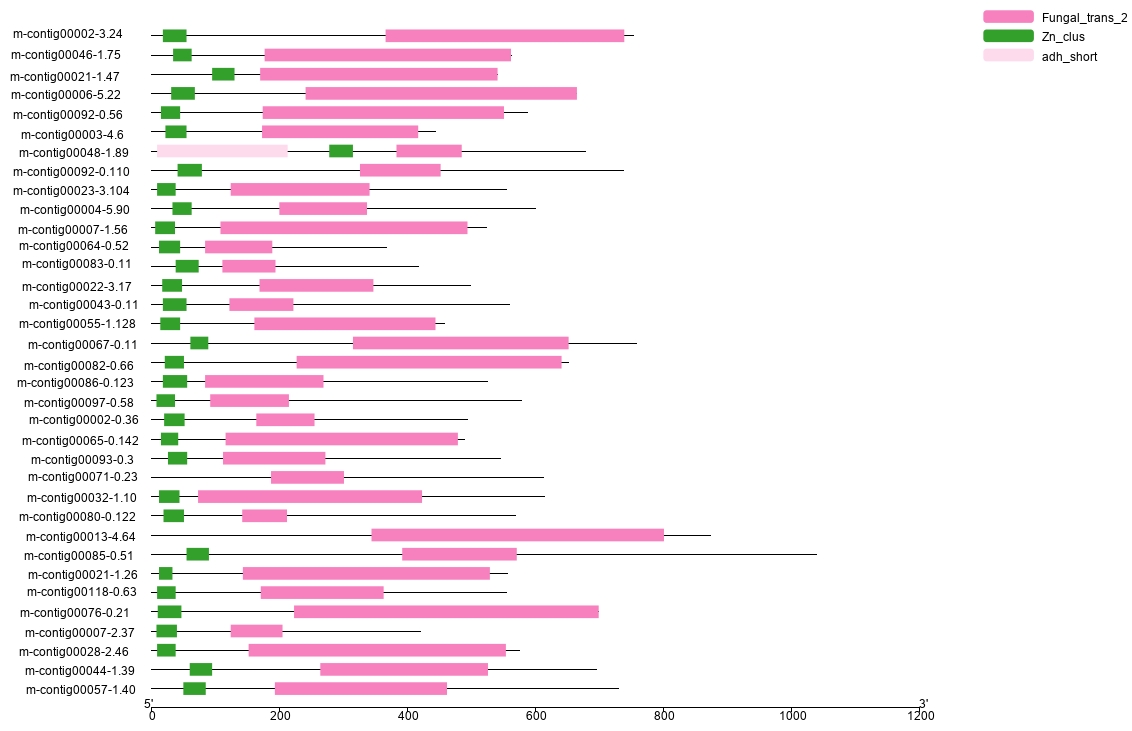


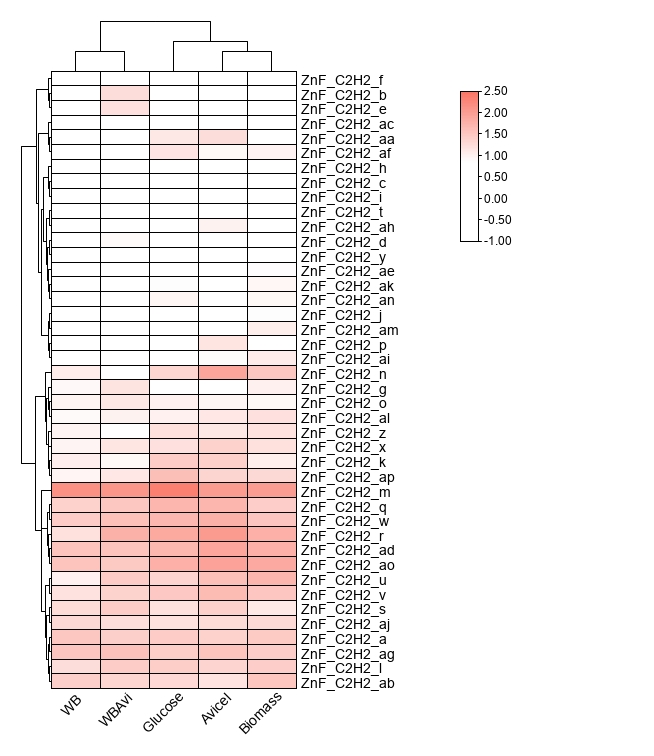

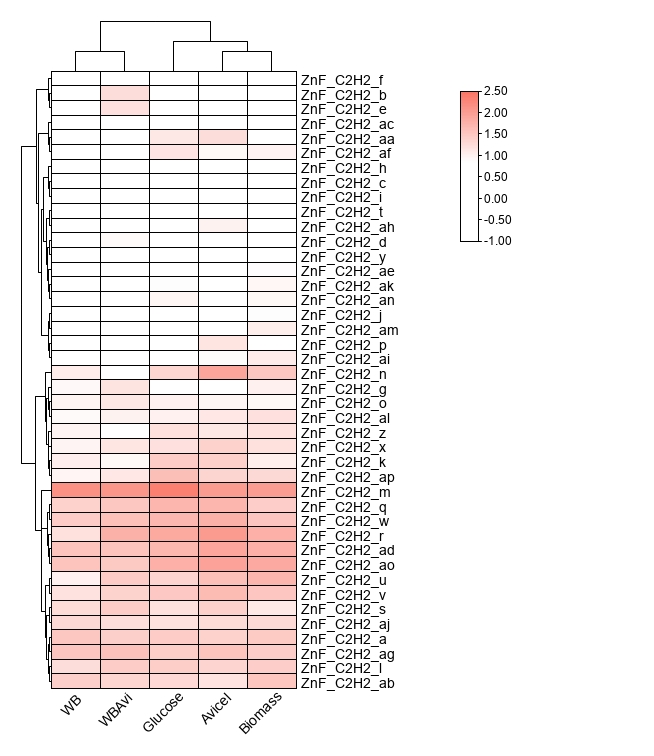

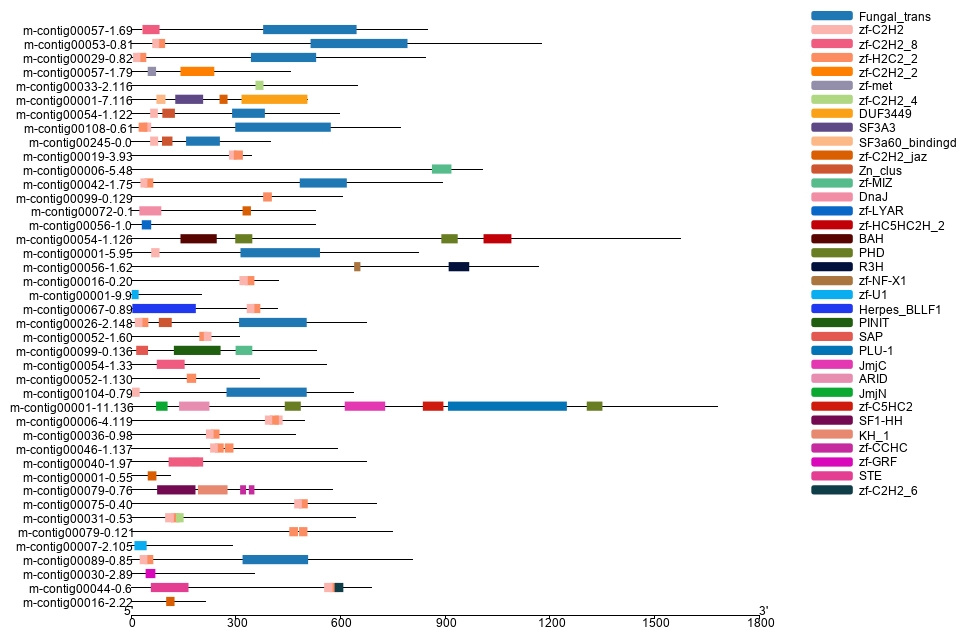

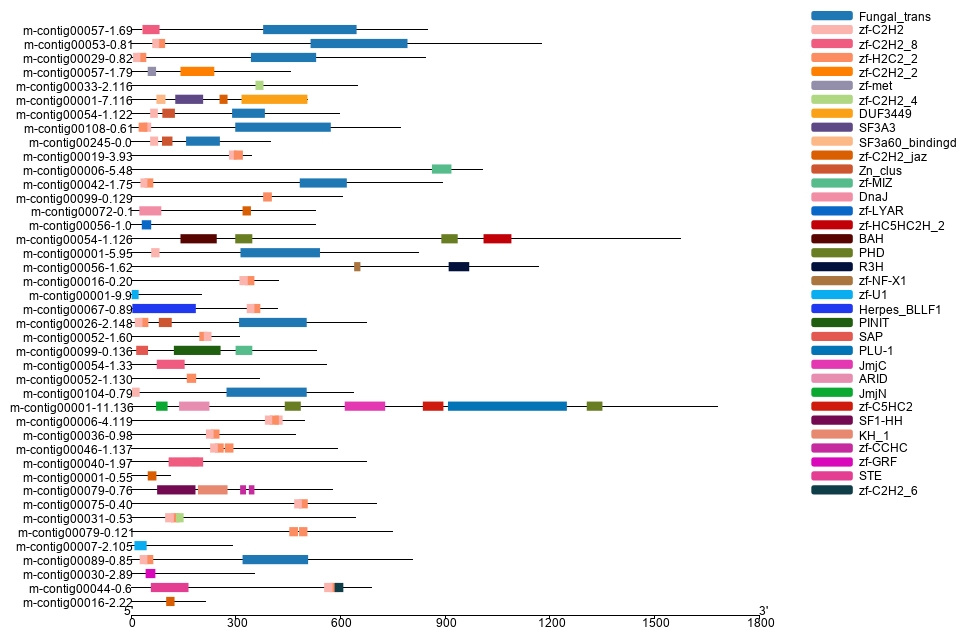


**F. ZnF_C2H2 family**

**Figure S2.** Expression analysis along with the domain architecture of *P. funiculosum* NCIM1228 TFs

**
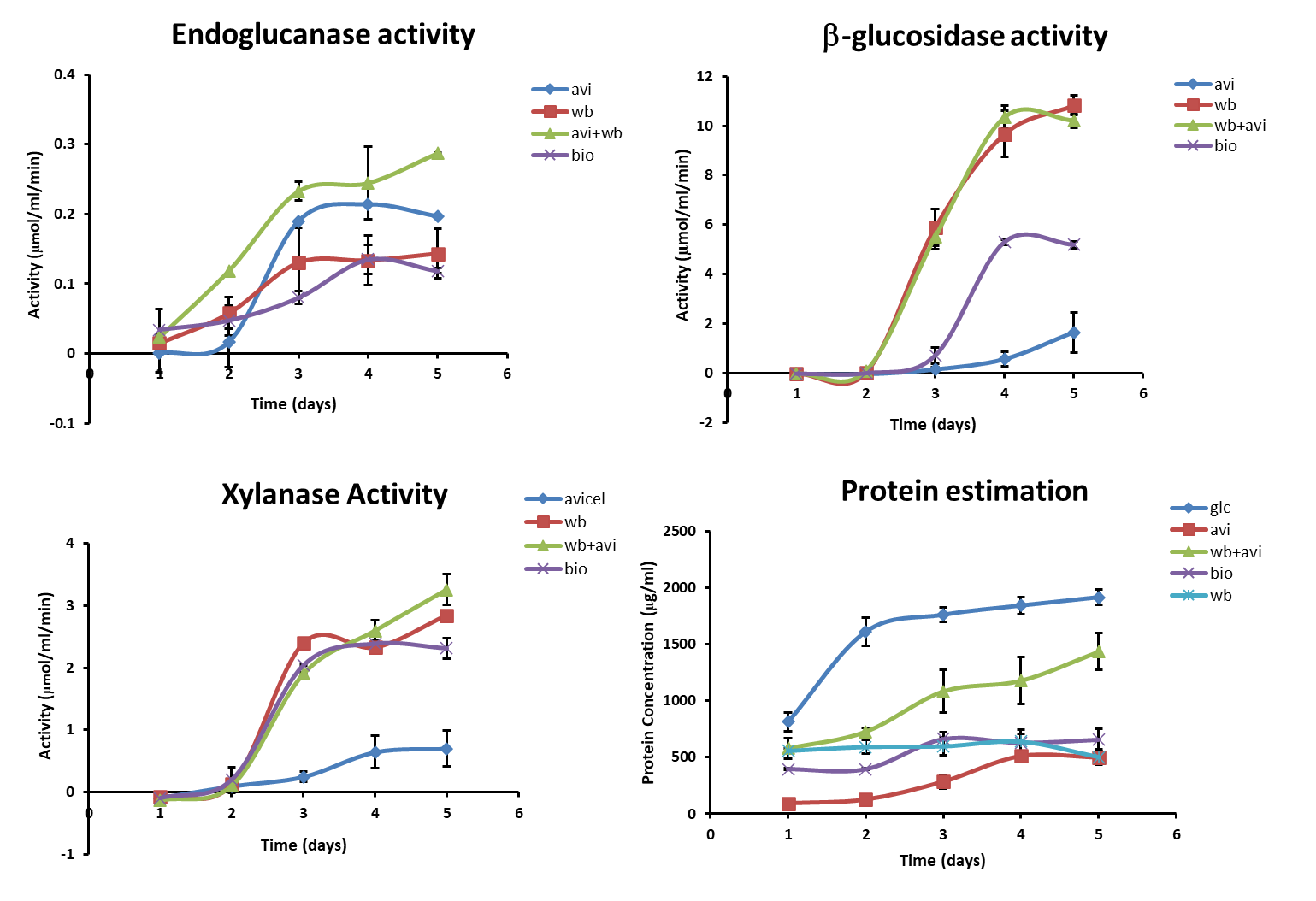
**

**Figure S3. Cellulolytic activities and supernatant protein estimation of *P. funiculosum* NCIM1228**


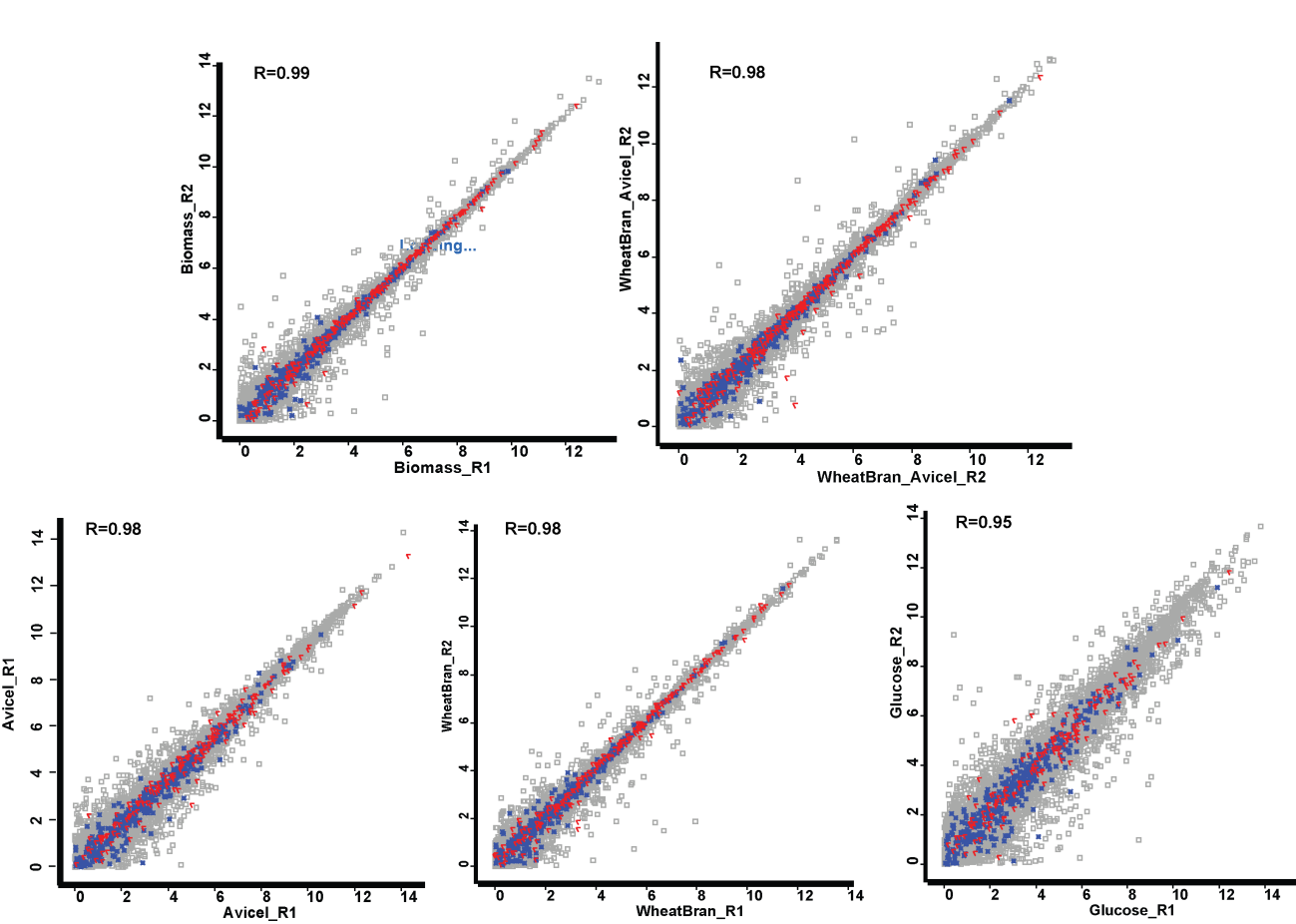

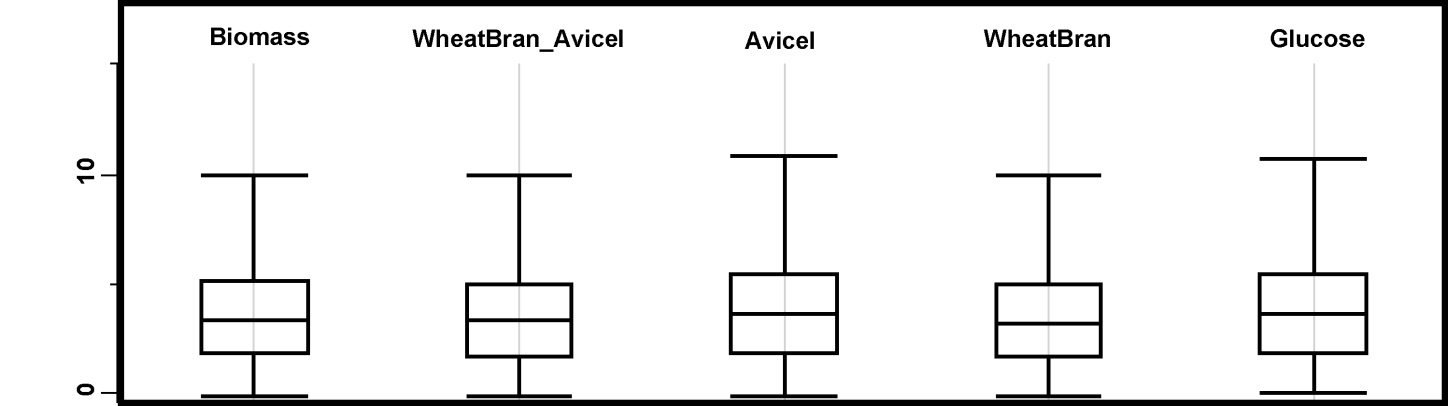


(B)

(A)

**Figure S4. Correlation of RNA-Seq Data obtained from the biological replicates. (A)** Graphs representing the Pearson correlation between biological replicates of each sample. A high Pearson correlation was obtained demonstrating the reliability of RNA-seq analysis (R≥0.95). **(B)** Boxplot of all normalized samples showing that all samples and conditions are comparable.

**
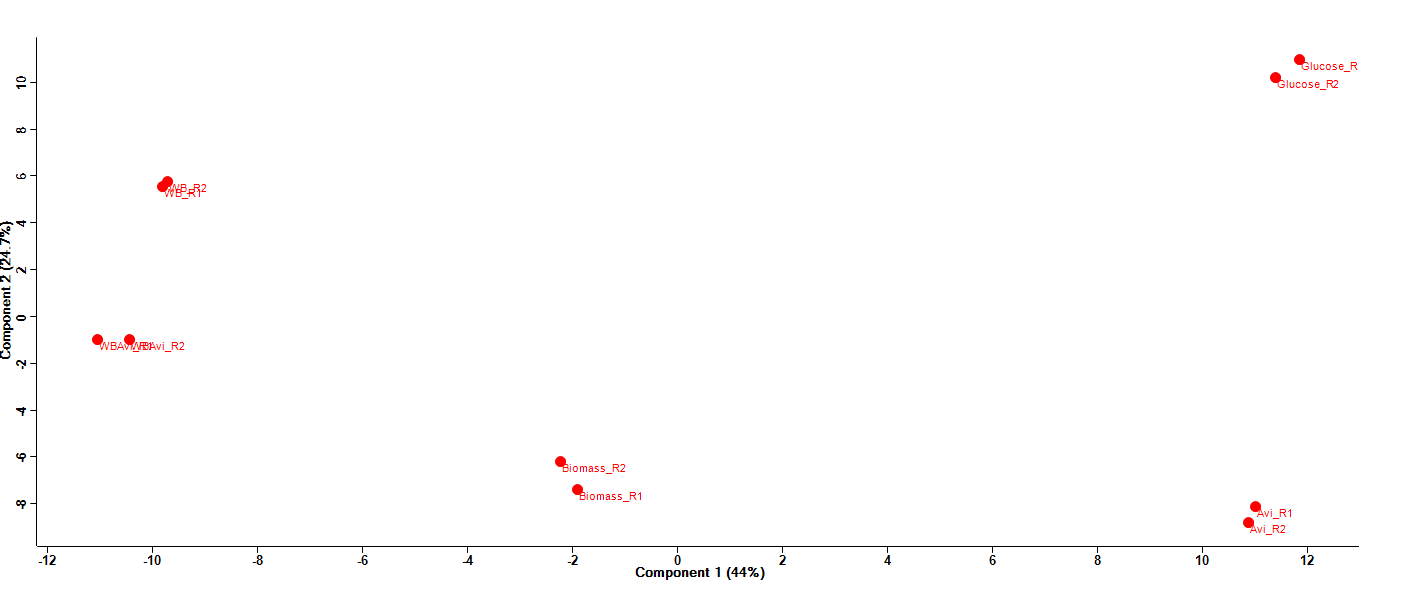

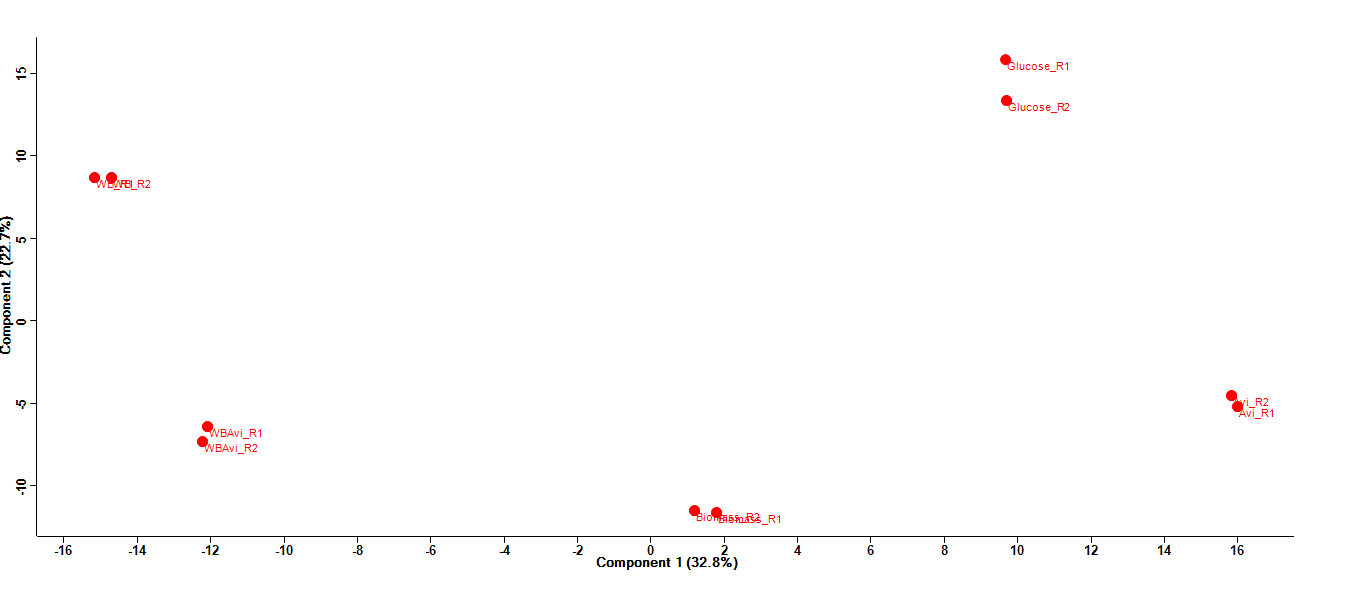
(A)**

**(B)**

**Figure S5.** Principal component analysis (PCA) of gene expression levels in A) CAZymes and B) TFs in replicates of the five carbon substrates-Glucose, Avicel, Wheat bran, Avicel + wheat bran, and biomass.


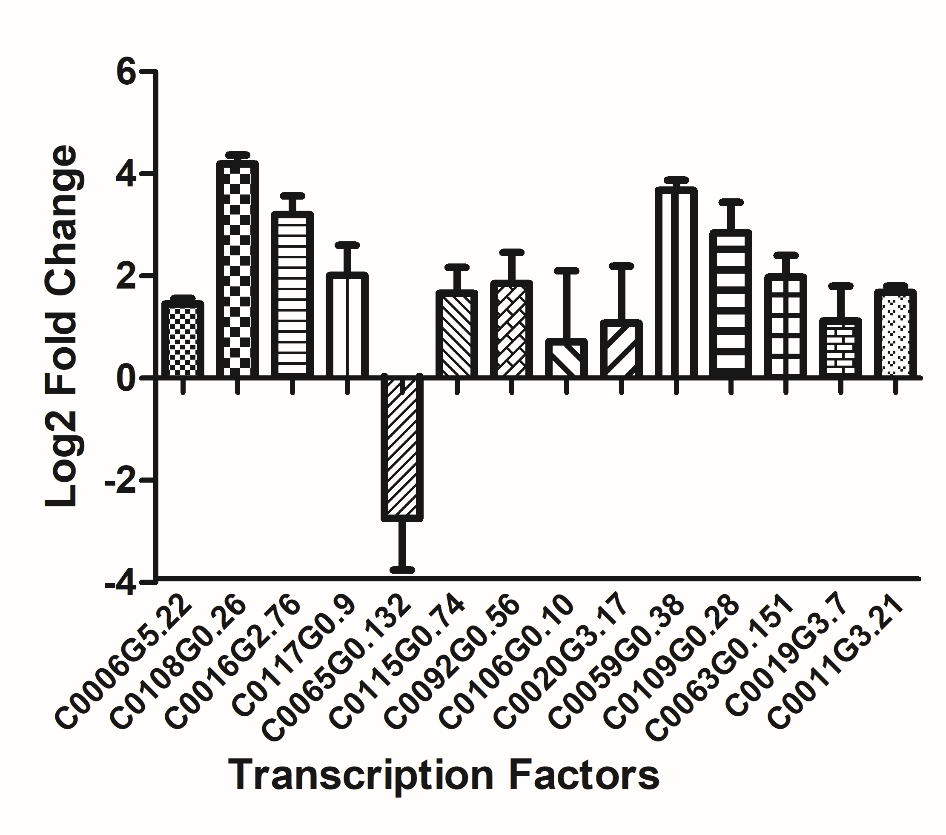


**Figure S6. Expression analysis of TF coding genes in response to crystalline carbon substrate Avicel using RT-qPCR.** The X-axis represents the TF coding genes, and Y-axis indicates the relative expression level in Avicel wrt glucose. Relative fold change expression was calculated by ΔΔC_t_ method w.r.t. to an endogenous control Actin. Each bar represents an average of three biological replicates. Error bars indicate the + standard error.

**Figure S7.** Alignment of transcription factors with the known homologs.

**Alignment of C0016G2.76 with Transcription factor atf21 [*Talaromyces marneffei* PM1]**

Query 40 ISGIPPSSPLQHDELSFEHFIPPT-DQMDSPSSITSPDFSLAQPDSHNNSYSSKKEKSRK 98

G+PPSSP QHDELSFEHFIPPT DQMDSPSSITSPDFS++Q D HN+ SYSSKKEKSRK

Sbjct 24 FKGMPPSSPPQHDELSFEHFIPPTTDQMDSPSSITSPDFSVSQRDGHNSSYSSKKEKSRK 83

Query 99 SRSAKSPASEEDTPENKSRRRREQNRIAQRTFRERKDRYIQNLESHIKLLDASHKDLQAS 158

SRS KSPASEEDTPENKSRRRREQNRIAQRTFRERKDRYIQNLESHIKLLDASHKDLQAS

Sbjct 84 SRSNKSPASEEDTPENKSRRRREQNRIAQRTFRERKDRYIQNLESHIKLLDASHKDLQAS 143

Query 159 YRQSTDQVNALYAQLLETQGELDYWRCLAQP--SATPTTTTAAAMTSTPTIASPVEAHGR 216

YRQSTDQVNALY QLLETQGELDYWRCLAQP S T TTTT M++TPTIA P EAHGR

Sbjct 144 YRQSTDQVNALYTQLLETQGELDYWRCLAQPSTSQTTTTTTTETMSATPTIA-PGEAHGR 202

Query 217 RH-HQNVPVMAGMHIPHGQFT 236

RH H N P+M+ GM+I HGQFT

Sbjct 203 RHLHPNAPIMSGMNIQHGQFT 223

**Alignment of C0006G5.22 with transcription factor NosA [*Talaromyces stipitatus* ATCC 10500]**

Query 1 MARPSAKRSSASSSRASAQDSERHHKRSRDGCYTCRLRRKKCDESHPSCRACIHLGVKCQ 60

MARP+ KRSSASSSRAS QD++R HKRSRDGCYTCRLRRKKCDE HPSCRACIHLGVKCQ

Sbjct 11 MARPATKRSSASSSRASVQDADRQHKRSRDGCYTCRLRRKKCDEGHPSCRACIHLGVKCQ 70

Query 61 YSKPSWWSNIEQRKVQKERLKNKIKQTKLNEKNNSIPGQRNRPLPLPLSSPYSPVDNFNR 120

Y+KPSWWSNIEQRKVQKERLKNKIKQTKLNEKNNSIPGQRNRPLPLPLSSPYSPV +FNR

Sbjct 71 YTKPSWWSNIEQRKVQKERLKNKIKQTKLNEKNNSIPGQRNRPLPLPLSSPYSPVHDFNR 130

Query 121 PFFPGTPIDPFGSQLPTPALGSTQFGQMGPYEVDVRTERQLYVNDVPTRIDTSFSTFNTF 180

PFFPGTP+DPFGSQLPTPAL TQFGQ+ GPYEVDVRTERQLYVNDVPTRIDTSFSTFNTF

Sbjct 131 PFFPGTP LDPFGSQLPTPALAPTQFGQLGPYEVDVRTERQLYVNDVPTRIDTSFSTFNTF 190

Q 81 VPPQMHATLPHMPNDEWLPPVTHSQVQFPYQPMGPMQFVPDGQSFCQMQLSIPVLDADRP 240

+PPQMHATLPHMPNDEWLPPV + VQFPY MGPM FVP+GQSF QMQLSIPVLDADRP

S 191 MPPQMHATLPHMPNDEWLPPVGQAPVQFPYHGMGPMPFVPEGQSFSQMQLSIPVLDADRP250

Query 241 LLNHFVEKVLRLIFPILEVHQKGHERAQAILQSLETNKSYLHCCLSVAAIHVKSTVGVIT 300

LLNHFVEKVLRLIFPILEVHQKGHERAQ+ILQSLETNKSYLHCCLSVAAIHVKSTVGVI

Sbjct 251 LLNHFVEKVLRLIFPILEVHQKGHERAQSILQSLETNKSYLHCCLSVAAIHVKSTVGVIN 310

Query 301 EEVDHDIMRHRYEAVAQLCKSLNEDSKHEQILEATLAMILFHCSVGGPDDHLPDIAWNDH360

EEVDHDIMRHRYEAVAQLC+SLNEDS+HEQILEATLAMILFHCSVGGPDDHLPDIAWNDH

Sbjct 311 EEVDHDIMRHRYEAVAQLCQSLNEDSQHEQILEATLAMILFHCSVGGPDDHLPDIAWNDH370

Query 361 FHAATSLINKLELPNALVQSNNNTVQPPFNMTLASWIDILGATMIGKSPQFAHTYRTKHL 420

FHAATSLINKLELPNALVQSNNNTVQPPFNMTLASWIDILGATMIGKSPQFAHTYRTKHL

Sbjct 371 FHAATSLINKLELPNALVQSNNNTVQPPFNMTLASWIDILGATMIGKSPQFAHTYRTKHL 430

Query 421 SGTSSGLRELMGCDDRVMYLISEIACLDALKAEGLVDDISLCSHVSALGQQLDATDYVDK 480

SGTSSGLRELMGCDDRVMYLISEIACLDALK EGLVDD+SLCSHVSALGQQLDATDYVDK

Sbjct 431 SGTSSGLRELMGCDDRVMYLISEIACLDALKTEGLVDDLSLCSHVSALGQQLDATDYVDK 490

Query 481 SLEHPYSALSGGIRPDQLTKNMTAIFRLAARIYLCSLVPGFDRNQASNVNLVDAVRYALQ 540

SLEHPYSALSGGIRPDQLTKNMTAIFRLAARIYLCSLVPGFDRNQASNVNLVDAVRY+LQ

Sbjct 491 SLEHPYSALSGGIRPDQLTKNMTAIFRLAARIYLCSLVPGFDRNQASNVNLVDAVRYSLQ 550

Query 541 FIPSGPDGYDRSLVWPLLITGAFSTPFSPFRHTLQSRIVEMGDQAEFGSFGRMYRVLEEV 600

FIPSGPDGYDRSLVWPLLITGAFSTPFSPFRHTLQSRI+EMGDQAEFGSFGRMYRVLEEV

Sbjct 551 FIPSGPDGYDRSLVWPLLITGAFSTPFSPFRHTLQSRIMEMGDQAEFGSFGRMYRVLEEV 610

Query 601WRLSDDPITPIPGLDGTLPSPTFPTPGNDVPISPTGSVSGMRELKKRDVHWRDVMTRNGW660

WRLSDDP+TPIPGLDGTLPSPTFPT GND+P+SPTGSVSGMRELKKRDVHWRDVMTRNGW

Sbjct 611WRLSDDPVTPIPGLDGTLPSPTFPTSGNDLPVSPTGSVSGMRELKKRDVHWRDVMTRNGW670

Query 661 KYLLI 665

KYLLI

Sbjct 671 KYLLI 675

**Alignment of C0108G0.26 with transcription factor (Fcr1)[*Talaromyces marneffei* ATCC 18224]**

Query 1 MAARQSTPSSEHSAHSDSNVRKRVCKACDRCRLKKSKCDGASPCGRCRADNAICVFGERK 60

MAARQS+PSSEHSAHSDSNVRKRVCKACDRCRLKKSKCDGASPCGRCRADNAICVFGERK

Sbjct 1 MAARQSSPSSEHSAHSDSNVRKRVCKACDRCRLKKSKCDGASPCGRCRADNAICVFGERK 60

Query 61 KAHDKVYPKGYVEMLEQQQIWLVNGLQELYRRAQEGEGWTGEPLKAESNGHPLTHDLLTR 120

KAHDKVYPKGYVEMLEQQQIWLVNGLQELYRRAQEGEGWTGEPLKAESNGHPLTHDLLTR

Sbjct 61 KAHDKVYPKGYVEMLEQQQIWLVNGLQELYRRAQEGEGWTGEPLKAESNGHPLTHDLLTR 120

Query 121 LGALDHTKGETFEDNVEQMQQRLWQQNAGLMQRQESSDGSSDIAHSPPT 169

LGALDHTKGE FEDNVEQMQQ+LWQQNAGLMQRQESSDGSSDIAHSPPT

Sbjct 121 LGALDHTKGEIFEDNVEQMQQKLWQQNAGLMQRQESSDGSSDIAHSPPT 169

**References**

39. Li, C.-X., et al., *Genome sequencing and analysis of Talaromyces pinophilus provide insights into biotechnological applications*. Scientific Reports, 2017. **7**(1): p. 490.

52. Van Den Berg, M.A., et al., *Genome sequencing and analysis of the filamentous fungus Penicillium chrysogenum.* Nature biotechnology, 2008. **26**(10): p. 1161-1168.

53. Fujii, T., et al., *Draft genome sequence of Talaromyces cellulolyticus strain Y-94, a source of lignocellulosic biomass-degrading enzymes.* Genome Announcements, 2015. **3**(1): p. e00014-15.

54. Berka, R.M., et al., *Comparative genomic analysis of the thermophilic biomass-degrading fungi Myceliophthora thermophila and Thielavia terrestris.* Nature biotechnology, 2011. **29**(10): p. 922-927.

55. Li, W.-C., et al., *Trichoderma reesei complete genome sequence, repeat-induced point mutation, and partitioning of CAZyme gene clusters.* Biotechnology for biofuels, 2017. **10**(1): p. 1-20.

56. Koike, H., et al., *Comparative genomics analysis of Trichoderma reesei strains.* Industrial Biotechnology, 2013. **9**(6): p. 352-367.

57. Christopher, M., et al., *Early cellular events and potential regulators of cellulase induction in Penicillium janthinellum NCIM 1366.* Scientific Reports, 2023. **13**(1): p. 5057.

58. Peng, M., et al., *The draft genome sequence of the ascomycete fungus Penicillium subrubescens reveals a highly enriched content of plant biomass related CAZymes compared to related fungi.* Journal of biotechnology, 2017. **246**: p. 1-3.

59. Mardones, W., et al., *The genome sequence of the soft-rot fungus Penicillium purpurogenum reveals a high gene dosage for lignocellulolytic enzymes.* Mycology, 2018. **9**(1): p. 59-69.

60. Liu, G., et al., *Genomic and secretomic analyses reveal unique features of the lignocellulolytic enzyme system of Penicillium decumbens.* PloS one, 2013. **8**(2): p. e55185.
